# Brain-protective mechanisms of autophagy associated circRNAs: Kick starting self-cleaning mode in brain cells *via* circRNAs as a potential therapeutic approach for neurodegenerative diseases

**DOI:** 10.3389/fnmol.2022.1078441

**Published:** 2023-01-16

**Authors:** Rabea Basri, Faryal Mehwish Awan, Burton B. Yang, Usman Ayub Awan, Ayesha Obaid, Anam Naz, Aqsa Ikram, Suliman Khan, Ijaz ul Haq, Sadiq Noor Khan, Muslim Bin Aqeel

**Affiliations:** ^1^Department of Medical Lab Technology, The University of Haripur (UOH), Haripur, Pakistan; ^2^Sunnybrook Health Sciences Centre, Sunnybrook Research Institute, Toronto, ON, Canada; ^3^Department of Laboratory Medicine and Pathobiology, University of Toronto, Toronto, ON, Canada; ^4^Institute of Medical Sciences, University of Toronto, Toronto, ON, Canada; ^5^Institute of Molecular Biology and Biotechnology (IMBB), The University of Lahore (UOL), Lahore, Pakistan; ^6^Department of Public Health and Nutrition, The University of Haripur (UOH), Haripur, Pakistan

**Keywords:** circRNAs, autophagy, neurodegeneration, nervous system, therapeutics

## Abstract

Altered autophagy is a hallmark of neurodegeneration but how autophagy is regulated in the brain and dysfunctional autophagy leads to neuronal death has remained cryptic. Being a key cellular waste-recycling and housekeeping system, autophagy is implicated in a range of brain disorders and altering autophagy flux could be an effective therapeutic strategy and has the potential for clinical applications down the road. Tight regulation of proteins and organelles in order to meet the needs of complex neuronal physiology suggests that there is distinct regulatory pattern of neuronal autophagy as compared to non-neuronal cells and nervous system might have its own separate regulator of autophagy. Evidence has shown that circRNAs participates in the biological processes of autophagosome assembly. The regulatory networks between circRNAs, autophagy, and neurodegeneration remains unknown and warrants further investigation. Understanding the interplay between autophagy, circRNAs and neurodegeneration requires a knowledge of the multiple steps and regulatory interactions involved in the autophagy pathway which might provide a valuable resource for the diagnosis and therapy of neurodegenerative diseases. In this review, we aimed to summarize the latest studies on the role of brain-protective mechanisms of autophagy associated circRNAs in neurodegenerative diseases (including Alzheimer’s disease, Parkinson’s disease, Huntington’s disease, Spinal Muscular Atrophy, Amyotrophic Lateral Sclerosis, and Friedreich’s ataxia) and how this knowledge can be leveraged for the development of novel therapeutics against them. Autophagy stimulation might be potential one-size-fits-all therapy for neurodegenerative disease as per considerable body of evidence, therefore future research on brain-protective mechanisms of autophagy associated circRNAs will illuminate an important feature of nervous system biology and will open the door to new approaches for treating neurodegenerative diseases.

## 1. Introduction

Circular RNAs (circRNAs) are a novel recognized class of endogenous single stranded regulatory noncoding RNAs (ncRNAs) characterized by forming circular transcripts (backsplicing) with neither 5′-to-3′ polarity nor a polyadenylated tail (Memczak et al., 2013). CircRNAs exhibit cell-specific, tissue-specific and developmental stage specific expression patterns with high stability due to the lack of free ends typically targeted by 3′ and 5′ exoribonucleases and have a half-life of more than 48 h compared to linear transcripts with half-life of ~6 h (Huang et al., 2017b). Furthermore, circRNAs exhibits distinct disease-specific characteristics under different pathological states which makes them promising therapeutic targets as well as potential diagnostic, predictive, and prognostic biomarkers for many incapacitating human diseases (Verduci et al., 2021; Zhao et al., 2022). Numerous ncRNAs have been reported to regulate cell death processes including autophagy, apoptosis and necrosis. Among reported ncRNAs, circRNAs have been reported to play key role in the regulation of autophagy either by affecting the expression of key autophagy proteins or by affecting the inhibition or activation of signaling pathways regulating autophagy (Wang et al., 2022). A number of studies have shown that there is a significant link/ correlation between circRNAs and autophagy (Zhang et al., 2017).

Autophagy is a fundamental biological process and a core molecular pathway that significantly affects nearly all aspects of human health and diseases, especially in neurodegenerative diseases (Yang and Klionsky, 2020; Klionsky et al., 2021). Being a catabolic process, autophagy ensures neuronal health through preventing cell toxicity by removing long-lived proteins or defective organelles in the central nervous system (Filippone et al., 2022). There is accumulating evidence that autophagy pathways are deregulated in neurodegenerative diseases which contributes to the formation and accumulation of misfolded protein aggregates (a hallmark shared by several neurodegenerative diseases; Filippone et al., 2022). During this highly conserved cellular degradation process, organelles and portions of cytosol are sequestered into double membrane vesicles called autophagosome which then fuses with the lysosome for degradation by lysosomal hydrolases, therefore ensuring cellular and tissue level homeostasis under physiological and pathological conditions (Mizushima, 2007). Research efforts have focused heavily on developing strategies to facilitate the autophagic clearance of protein aggregates for therapeutic purposes in neurodegenerative disorders (Chandran and Rochet, 2022). Abnormal protein accumulation in nerve cells harbors a direct link with impaired macroautophagy (Yang and Klionsky, 2020). In one of the study, authors reported that autophagy associated circRNA may delay senile dementia which may prevent or delay the progress of AD suggesting that enhanced autophagy could be a potential therapeutic strategy for AD (Diling et al., 2019). Compared to cancers, research on the roles of autophagy in neurodegenerative diseases is still a nascent area of research, as relevant clinical investigations and preclinical studies are lagging far behind. There are still lot of unanswered questions between autophagy and some of the most common neurodegenerative diseases which warrants further in-depth clinical investigations.

**Figure 1:**
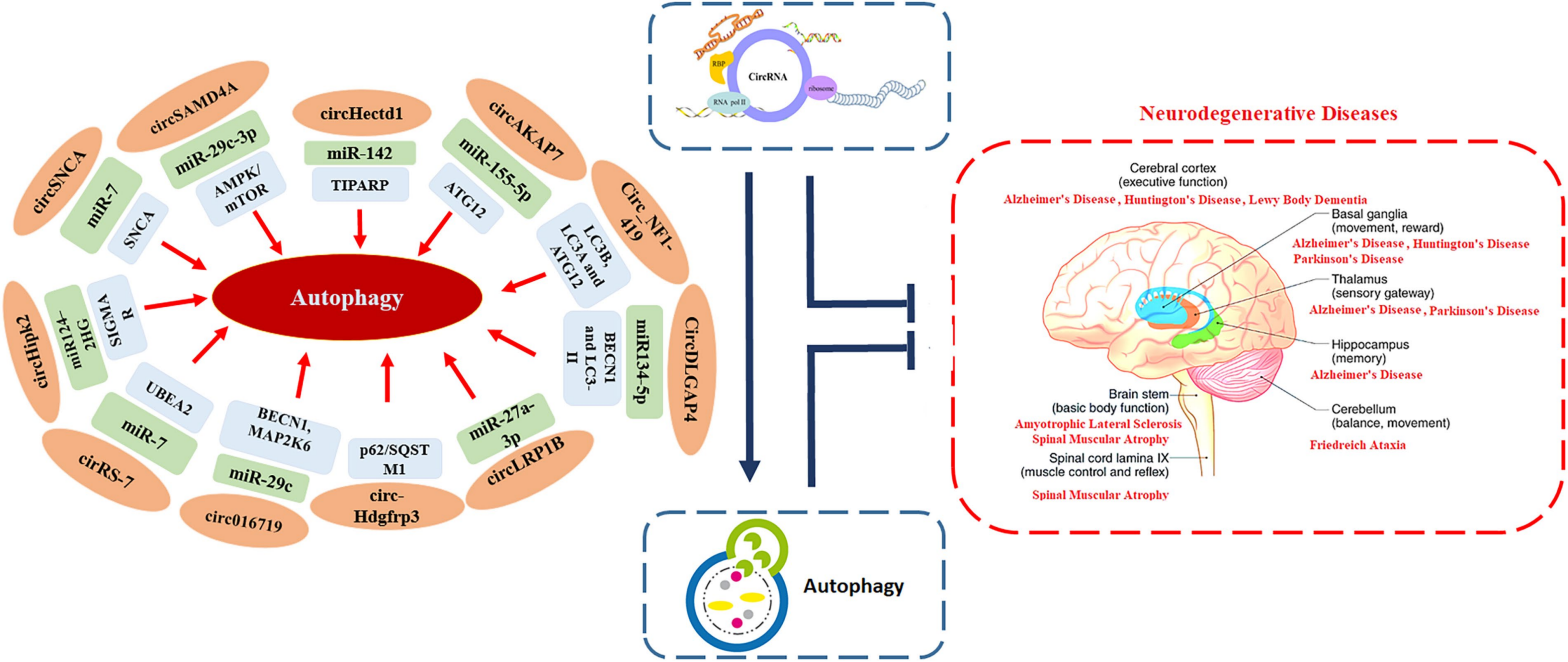
The interplay between circRNAs, autophagy and neurodegeneration.

Being an evolutionary conserved catabolic process that helps in recycling cellular components and damaged organelles, autophagy also enables cells to adapt to stress and changes in the internal and external environments (Wang et al., 2022). A number of studies have highlighted the crucial role of autophagy in regulating several pathological and physiological processes that are also regulated by circRNAs, such as cardiovascular diseases, autoimmune disorders, cancers, and neurodegenerative diseases (Figure 1; Wang et al., 2022). This suggests that, there may be regulatory crosstalk between autophagy and circRNAs in the development and progression of these human diseases (Zhou et al., 2021b).

CircRNAs have been reported to be unusually enriched in the nervous system (with continuous increase from the embryonic to the adult stage) especially in synapses with important regulatory roles in various brain functions (Sekar and Liang, 2019; Chen et al., 2022). CircRNA CDR1 (ciRS-7) was the first circRNA discovered in the human hippocampal CA1 formation. Later on researchers revealed the significance of cirRS-7/miR-7/UBEA2 axis in AD. They reported that down-regulation of ciRS-7 in AD contributed to an up-regulation of miRNA-7 which in turn down-regulated an autophagic, phagocytic protein essential in the clearance of neurotoxic amyloid-beta (Aβ) peptides namely ubiquitin protein ligase A (UBE2A; Zhao et al., 2022). It has been suggested that the deregulation of circRNA signaling is involved in blood–brain barrier disruption, angiogenesis, inhibition of pro-inflammatory signaling, apoptosis, autophagy disruption and alteration of cognitive functions in both acute and chronic CNS injury (Zhao et al., 2022). Regardless of the well-documented synaptic and neuronal circRNAs that display abnormalities in brain diseases, an ample understanding of the circRNA landscape and its downstream regulatory pathways in the human brain is still missing (Li et al., 2022c).

Among different risk factors reported so far, aging has been established as the most important and greatest known primary risk factor for the common neurodegenerative diseases such as Alzheimer’s disease (AD) and Parkinson’s Disease (PD; Gandhi and Abramov, 2012). Accumulating evidence supports a beneficial and direct role of autophagy in the aging process, with multiple genetic experiments revealing its significance in counteracting ageing and age-related diseases (Hansen et al., 2018). Research has shown that during brain aging, hundreds of circRNAs dramatically increase their expression profile compared to the host genes in various organisms (D’anca et al., 2022).

Oxidative stress is another well accepted risk factor inked with neuronal dysfunction and neurodegenerative diseases and has been reported to be involved in the propagation of neuronal injury (Paloczi et al., 2018). In-addition activation of astrocytes, the most abundant cell type in the central nervous system (CNS) has been reported to play detrimental role in various neurological pathologies, including stroke, PD and AD (Huang et al., 2017a). Previously reported research has demonstrated that activation of nuclear factor erythroid 2-related factor 2 (Nrf2), a ubiquitous master transcription factor that up-regulates antioxidant response elements (AREs) in astrocytes plays a neuroprotective role in both acute neuronal damage and chronic neurodegeneration-related oxidative stress (Zhang et al., 2013). In order to identify key circRNAs that regulate Nrf2-mediated neuroprotection in the brain Yang et al., performed microarray analysis of corpus striatum and substantia nigra in Nrf2 (−/−) and Nrf2 (+/+) mice. A total of 65 differentially expressed circRNAs were found in substantia nigra tissue whereas 50 significant differentially expressed circRNAs were obtained in corpus striatum tissue, respectively. Further analysis revealed 17 shared differentially expressed circRNAs in both tissues. The authors proposed that by sponging mmu-miR-34a and mmu-miR-27a, MmucircRNA-015216 might play a role in Nrf2 neuroprotection (Yang et al., 2018). Huang and co-authors revealed that specific blockage of circRNA circHIPK2 shows potential as a therapeutic target for a broad range of neuroinflammatory disorders through inhibition of astrocyte activation (Huang et al., 2017a).

Research has shown that cerebral ischemia is another one of the most common pathological factor in many neurodegenerative diseases and is being regulated by complex gene regulatory networks. Cerebral ischemia may lead to aggregation of neurodegeneration-related disease proteins including PSF/SFPQ, p54/NONO, TDP43, FUS, and hnRNPA1, all of which have been linked to neurodegeneration associated with frontotemporal dementia and amyotrophic lateral sclerosis (ALS; Kahl et al., 2018). In order to determine common regulators of cerebral and retinal neurodegeneration, Jiang et al., performed circRNA microarray profiling on C57BL/6J mice with transient middle cerebral artery occlusion. Analysis revealed 217 differentially expressed circRNAs. After in-depth analysis of 217 circRNAs, authors examined the role of circ-GLIS3 in neurodegeneration. Analysis of ischemia-induced neurodegenerative models unveiled significant up-regulation of circ-GLIS3. Authors concluded that circGLIS3 is a common regulator of neurodegeneration as well as a regulator of neuronal cell injury by acting as miR-203 sponge (Jiang et al., 2021). Extensive and progressive neurodegeneration has been observed in Ischaemic stroke survivors compared to stroke-free controls (Egorova et al., 2020).

Glial cells (non-neuronal cells) namely astrocytes, oligodendrocytes, and microglial cells exist throughout the mature central nervous system, and have been reported to play important role in regulating various aspects of neural development and function (including synaptogenesis and synaptic function; Liu et al., 2022). Glial cells constitute around 50% of the total brain volume and recently reported transcriptomics and genetic studies have strongly suggested that glia are the first cells changing with aging which is one of the most important most important risk factor for the common neurodegenerative diseases (Salas et al., 2020). In order to identify senescence-regulated astroglial circRNAs, Diling et al., generated D-galactose glial cell aging model. Results revealed significant number of differentially expressed circRNAs in astrocytes demonstrating their positive correlation with different degrees of aging. Authors selected circNF1-419 for further analysis because of having greatest differential expression and generated over-expressed circNF1-419-transfected rat astrocyte. Analysis revealed differential levels of LC3B, LC3A and ATG12 in overexpressed rat astrocyte compared to wild type astrocyte. Further *in-vivo* experiments in 12 months old Balb/c mice and 8 months old SAMP8 mice revealed that circNF1-419 accelerates the process of autophagy by binding to Adaptor protein 2 B1 and Dynamin-1 proteins. The authors concluded that circNF1-419 may defer senility and therefore delay the progress of AD, further suggesting that enhanced autophagy could be an effective therapeutic strategy for AD (Diling et al., 2019).

Diagnostic tools and therapeutic strategies based on autophagy associated circRNAs have a strong and currently untapped potential role in neurodegenerative diseases. Further in-depth research on regulatory roles of autophagy associated circRNAs can identify multiple innovative epigenetic and molecular biomarkers for age-related neurodegenerative disorders. Meanwhile no effective treatment strategy exists for a number of neurodegenerative diseases which should serve as a natural driver for the implementation of these types of circRNA-mediated therapies. Moreover the success rate of neurological drug trials to date is very low and trails have not revealed any promising results. In addition, progress on identifying novel candidates that potentiates autophagy has been limited by the drawbacks of existing assays designed to monitor autophagic flux.

In this review, we aimed to summarize the latest studies on the role of brain-protective mechanisms of autophagy associated circRNAs in neurodegenerative diseases (including AD, PD, Huntington’s disease (HD), Spinal Muscular Atrophy (SMA), ALS, and Friedreich’s ataxia (FRDA)), and how this knowledge can be leveraged for the development of novel therapeutics against them. Autophagy stimulation might be potential one-size-fits-all therapy for neurodegenerative disease as per considerable body of evidence, therefore future research on brain-protective mechanisms of autophagy associated circRNAs will illuminate an important feature of nervous system biology and will open the door to new approaches for treating neurodegenerative diseases.

## 2. The enigmatic connection between circRNAs, autophagy, and neurodegeneration

### 2.1. Interplay between circRNAs, autophagy, and AD

AD is a progressive neurological condition associated with aging, and is the primary cause of dementia in humans, comprising around 60–80% of cases (Crous-Bou et al., 2017). By 2050, it is estimated that the global incidence of people with AD is set to increase to more than 100 million (Lynch, 2020). AD was first described in 1906; despite decades of study, the molecular basis of AD’s pathogenesis remains a mystery. In contrast, no well-documented therapeutic approaches exists which can slow or arrest the disease’s advancement. Multiple risk factors, such as aging, genetic predisposition, hereditary, occupation, and neurological damage, contributes to AD development (Association, A. S, 2010). AD is defined by developing distinct protein aggregates namely extracellular amyloid-beta (Aβ) plaques formation and intracellular neurofibrillary tangles (NFTs) formation. As the condition worsens, afflicted brain areas succumb to toxic stress, as indicated by massive neuronal death and CNS shrinkage (Querzfurth, 2010). Studies have revealed that autophagy is a primary modulator of Aβ generation and clearance (Nilsson and Saido, 2014). In addition, there is substantial evidence that failure in autophagy process is related to AD and other neurodegenerative disorders which leads towards gradual accumulation of misfolded proteins particularly Aβ aggregates (Oddo et al., 2006). In contrast, in healthy brain tissues, the synthesis of Aβ peptides is substantially lesser compared to the clearance rate, at 7.6 and 8.2% per hour, respectively (Bateman et al., 2006).

Aβ peptides are produced by the autophagosomal cleavage of amyloid precursor protein (APP) during the autophagic changeover of APP-rich organelles. In AD, autophagolysosome development and retrograde trafficking to the neural body are inhibited (Nixon, 2007), resulting in an enormous accumulation of autophagic vacuoles in neural cells. This accumulation might be associated with ESCRT-III malfunction, leading to neurodegeneration (Lee et al., 2007; Yamazaki et al., 2010), and may impair the fusing of autophagosomes with the endo-lysosomal system, hence affecting autophagosome differentiation (Rusten and Stenmark, 2009). The clearance of Aβ from the brain is accomplished by several mechanisms. First, they are susceptible to being destroyed directly by different Aβ-degrading proteases, such as BACE1 and CTSD (Saido and Leissring, 2012). Second, Aβ peptides aggregate in dystrophic neurites (the major elements of neuritic senile plaques in AD), becoming a significant source of the main intracellular pool of lethal peptides (Nixon et al., 2005; Yu et al., 2005). In addition, the recycling route of Aβ peptides is found in the neurons of patients with AD (Nilsson et al., 2013; Nilsson and Saido, 2014). According to a recently published study, neurons produce Aβ peptides in an autophagy-dependent way, and the deposition of intracellular Aβ aggregation is cytotoxic to CNS, enabling the development of AD (Nilsson et al., 2013). In conclusion, defective autophagy is a well-documented pathway in the pathophysiology of Aβ metabolism associated with AD.

AD primarily impacts the hippocampus, amygdala, and other parts of the brain (Association, A. S, 2019). It is commonly thought that Aβ deposition, tau protein NFTs, neurotoxicity, neurovascular dysfunction, and oxidative stress are involved in the etiology of AD (Youdim, 2010; Robinson et al., 2017). Reports have shown that circRNAs accumulate in the brains of aged people, and they are found in higher concentrations in the brains of mammals than in any other tissue (Rybak-Wolf et al., 2015; You et al., 2015). Some circRNAs accrue considerably in the brain subcellular compartments (such as synapses) throughout the course of neuronal ageing compared to their respective mRNAs; as a result, they might be considered as distinct class of ageing biomarkers (Gruner et al., 2016). In addition, ageing-related defective alternative splicing might result in an upsurge in neuronal circRNA biosynthesis (Fletcher et al., 2018). Synapses have the highest amount of circRNAs and their frequency rises with ageing, revealing they are involved in neurodevelopment and synaptic plasticity (Gruner et al., 2016). Various species accumulate circRNA throughout ageing, suggesting it might be a harmful agent for ageing and age-related disorders, likewise AD (Cai et al., 2019; Lo et al., 2020). CircRNAs have been demonstrated to ameliorate AD-like clinical symptoms in cellular and animal models of the disease, highlighting their function in regulating AD progression (Dube et al., 2019).

**Figure 2:**
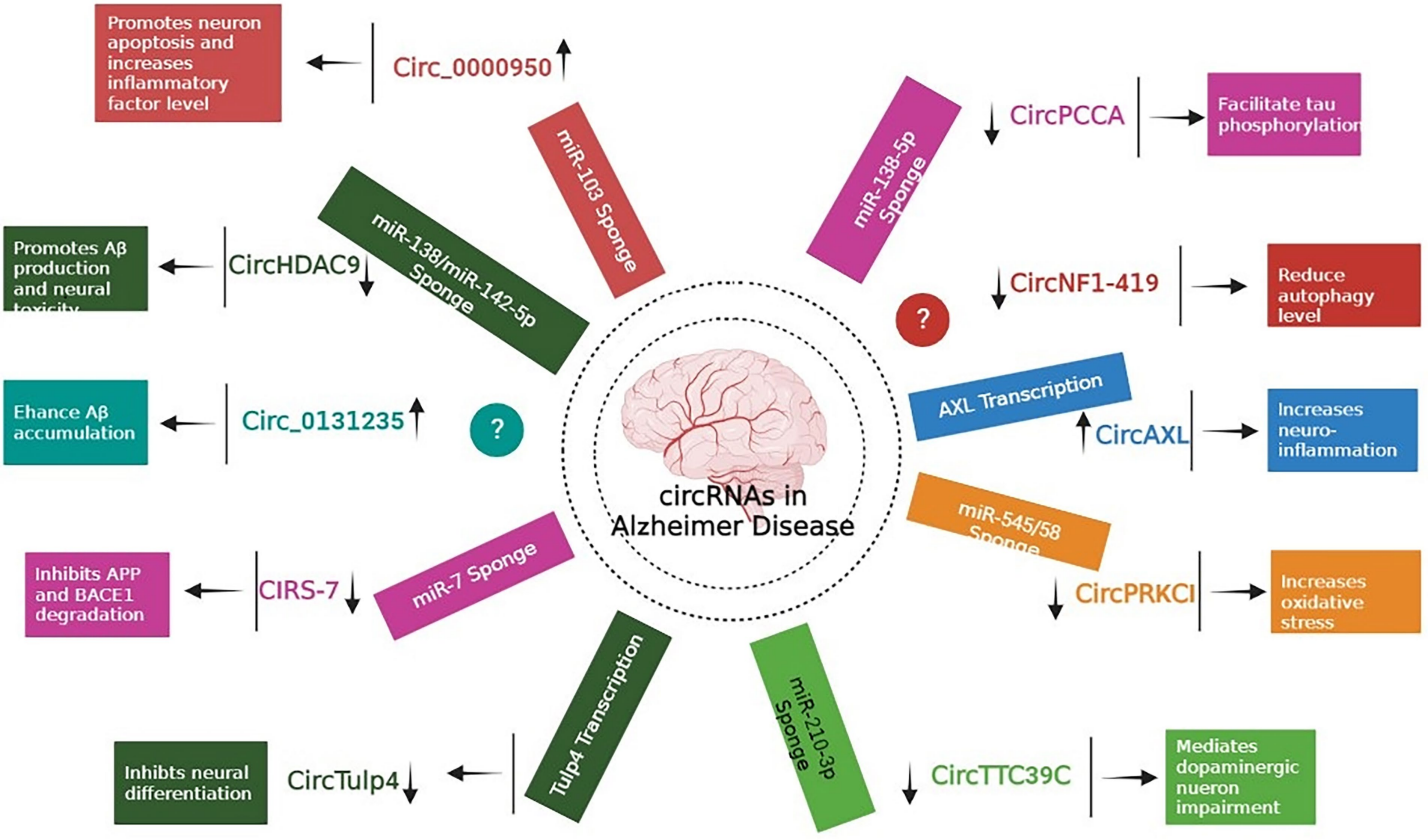
Role of different circRNAs in AD reported in both *In-vitro* and *In-vivo* models (Wang et al., 2015; Xie et al., 2016; Zhao et al., 2016; Ray et al., 2017; Shi et al., 2017; Zhang et al., 2018; Cheng et al., 2019; Diling et al., 2019; Lu et al., 2019; Yang H. et al., 2019; Li et al., 2020b; Bigarré et al., 2021; Ravanidis et al., 2021; Zhang and Bian, 2021).

Extensive research has focused predominantly on circRNAs, and it is becoming apparent that few of them (such as circRNA Cdr1as and circ 000950) act as microRNA sponges, disrupting downstream miRNAs and contributing to the development of neurodegenerative disorders (Piwecka et al., 2017; Wang et al., 2018). In one of the study, authors performed microarray analysis to study the expression pattern of circRNAs in SAMP8 mice (animal model of overproduction of APP and oxidative damage). Results revealed 85 differentially expressed circRNAs. One of the most significantly dysregulated circRNA, mmu_circRNA_017963, was found to be strongly related with autophagosome assembly which reveals possible implication of involvement of autophagy associated circRNA in AD (Figure 2; Huang et al., 2018; Tables 1 and 2).

**Table 1:** List of circRNAs reported to be associated with autophagy and neurodegeneration.

circRNAs	Neurodegenerative Diseases	Functions	References
circNF1-419	Alzheimer’s disease	Delays the progress of Alzheimer’s disease and defer senility	Diling et al., (2019)
circHDAC9	Alzheimer’s disease	Mediates synaptic and amyloid precursor protein processing deficits in Alzheimer’s disease	Lu et al., (2019)
ciRS-7	Alzheimer’s disease	Shuttle neurotoxic and immunogenic amyloid peptides into proteolytic pathways *via* regulating miR-7/UBE2A signaling circuit	Zhao et al., (2016)
hsa_circ_0131235	Alzheimer’s disease	Prevents damage from Aß aggregation	Bigarré et al., (2021)
mmu_circRNA_017963	Alzheimer’s disease	Associated with Alzheimer’s disease pathogenesis *via* regulating autophagosome assembly, apoptotic process, exocytosis, transport and RNA splicing	Huang et al., (2018)
hsa_circ_0023919	Amyotrophic lateral sclerosis	Function is unknown, may function *via* targetting miR-9	Dolinar et al., (2019)
hsa_circ_0063411	Amyotrophic lateral sclerosis	Function is unknown, may function *via* targetting miR-647	Dolinar et al., (2019)
hsa_circ_0088036	Amyotrophic lateral sclerosis	Function is unknown	Dolinar et al., (2019)
circ-Hdgfrp3	Amyotrophic lateral sclerosis	Function is unknown, found to be associated with aggregates containing ALS-related FUS P525L (highly pathogenic mutation)	D'Ambra et al., (2021)
circHIPK2	Huntington’s disease	Regulates astrocyte activation *via* targeting miR124–2HG-SIGMAR1 pathway	Huang et al., (2017a)
circHectd1	Huntington’s disease	Regulates astrocyte activation *via* targeting miR142-TIPARP signaling circuit	Han et al., (2018)
circ016719	Huntington’s disease	plays a critical role in neuron cell apoptosis *via* targeting miR-29c/Map2k6 signaling circuit	Tang et al., (2020)
circRNA Cdr1as	Neurodegenerative disorders	Regulates neuronal development *via* sponging miR-7	Piwecka et al., (2017) and Wang et al., (2018)
circ-GLIS3	Neurodegeneration	Regulates middle cerebral artery occlusion (MCAO)-induced cerebral neurodegeneration	Jiang et al., (2021)
circSNCA	Parkinson’s disease	Affects autophagy and cell apoptosis *via* regulating miR-7/SNCA signaling cascade	Sang et al., (2018)
circDLGAP4	Parkinson’s disease	Increases autophagy, inhibits apoptosis and exerts neuroprotective effects *via* modulating miR-134-5p/CREB pathway	Feng et al., (2020)
circSAMD4A	Parkinson’s disease	Associated with autophagy and apoptosis of dopaminergic neurons *via* modulating miR-29c-3p/AMPK/mTOR pathway	Wang et al., (2021b)
circSLC8A1	Parkinson’s disease	Might play a role in oxidative stress *via* sponging miR-128	Hanan et al., (2020)

**Table 2:** List of autophagy associated circRNAs and neurodegeneration associated circRNAs along with possible connection and correlation.

CircRNAs related to Autophagy	CircRNAs related to Neurodegeneration	Autophagy associated CircRNAs related to Neurodegeneration	Proposed Autophagy associated circRNAs related to Neurodegeneration	miRNA/mRNA Regulatory Axis	References
circMUC16 (Gan et al., 2020)	circMUC16 (Zhou et al., 2021b)		circMUC16	hsa-miR-199a-5p/DRAM1, WNT2 hsa-miR-183/ UVRAG, FOXO1, FOXO3 hsa-miR-138, /NLRP3, Sirt1, hsa-miR-200a/ ATG7, OGG1-2a, hsa-miR-132/Tau, EP300, Sirtuin-1 and FOXO1a, hsa-miR-141/BCL2, BDNF, and SIRT1, hsa-miR-199a/ p62, Sp1, LRRK2, hsa-miR-29a/ NAV3, Puma, Bim, Bak, or Bace1 and HO-1	Min et al., (2010); Shioya et al., (2010); Roshan et al., (2014); Huangfu et al., (2016); Ye et al., (2017); Delavar et al., (2018); Fan et al., (2018); Roser et al., (2018); Fu et al., (2019); Li et al., (2019b, 2020a,b); Miao et al., (2020); Feng et al., (2021); Liu et al., (2021) and Zhou et al., (2021a)
circ_NF1-419 (Wang et al., 2022)	circ_NF1-419 (Zhou et al., 2021b)	circ_NF1-419 (Wang et al., 2021a)		hsa-miR-149-5p/AKT1/mTOR	Zhang et al., (2019)
circDLGAP4 (Wang et al., 2022)	circDLGAP4 (Zhang and Bian, 2021)	CircDLGAP4 (Feng et al., 2020)		hsa-miR-143 /p53, p62 hsa-miR134-5p/ BECN1 and LC3-II	Bai et al., (2016) and Feng et al., (2020)
ciRS-7 (Meng et al., 2020)	ciRS-7 (Kondo et al., 2020)	ciRS-7 (Zhao et al., 2016)		hsa-miR-7/ LKB1-AMPK-mTOR, UBE2A	Gu et al., (2017) and Zhou et al., (2019)
	circHDAC9 (Zhang et al., 2020)	circHDAC9 (Lu et al., 2019)		hsa-miR-138, /NLRP3, Sirt1	Ye et al., (2017) and Feng et al., (2021)
circHectd1 (Wang et al., 2022)		circHectd1 (Han et al., 2018)		hsa-miR-1,256/CAB39, hsa-miR-133b/ PTBP1, EGFR, hsa-miR-142 /CAMK2A, ATG16L1, hsa-miR-137/NR4A2, CPLX1, NSF, SYN3 and SYT1	Smrt et al., (2010), Sugiyama et al., (2016), Lu et al., (2018), Xu et al., (2019), Yang Q. et al., (2019), Gupta et al., (2020) and Tian et al., (2020)
Cdr1-as (Wang et al., 2022)		Cdr1as (Piwecka et al., 2017, Wang et al., 2018)		Hsa-miR-1,270/ IRF8	Yang Q. et al., 2019 and Jiménez-Ortega et al., (2017)
circAkap7 (Wang et al., 2022)				Hsa-miR-155-5p/SOCS1, TNF-α, IL13Rα1, SHIP1, TNFSF10 and SOCS-3	Cardoso et al., (2012); Xu et al., (2020); Burgaletto et al., (2021); and Zingale et al., (2021)
	MmucircRNA-015216 (Yang et al., 2018)			mmu-miR-34a and mmu-miR-27a/ Nrf2	Yang et al., (2018)
	circGLIS3 (Jiang et al., 2021)			hsa-miR-203/Akirin2, Bcl2l2, Dgkb, Mapk10 and Vsnl1	Li et al., (2022a)
circLrp1b (Wang et al., 2022)		circLRP1B (Wang et al., 2022)		hsa-miR-27a-3p/GSK3ß, Wnt/ß-catenin, GLP1R	Zeng et al., (2021) and Harati et al., (2022)
	circ_0000950 (Zhang et al., 2020)			hsa-miR-103/ SOX2, Ndel1	Li et al., (2018)
Hsa_circ_0001546 (Ravanidis et al., 2021)				hsa-miRNA-421/ ATM/Chk2/p53	Wu et al., (2020)
circSNCA (Sang et al., 2018)				hsa-miR-7/SNCA	Sang et al., (2018)
	circRNA zip-2 (Kumar et al., 2018)			hsa-miR-60/ SNCA	Kumar et al., (2018)
circHipk2 (Wang et al., 2022)		circHIPK2 (Wang et al., 2022)		hsa-miR-124–2HG/ SIGMAR	Huang et al., (2017a)
		circAKAP7 (Xu et al., 2020)		hsa-miR-155-5p/ ATG12	Xu et al., (2020)
		circSAMD4A (Wang et al., 2021b)		hsa-miR-29c-3p/ AMPK/mTOR hsa-miR-124/ AMPK/mTOR	Wang et al., (2021b)
		circSLC8A1 (Hanan et al., 2020)		hsa-miR-128/AEG-1	Cao et al., (2020) andHanan et al., (2020)
		circ016719 (Tang et al., 2020)		hsa-miR-29c/ MAP2K6, BECN1	Tang et al., (2020)

Accumulation of Aβ proteins is an initial pathological feature of AD, which is exacerbated by the faulty production of APP and the abnormal level of BACE1 (Alcendor, 2020). Research revealed that miR-138 levels in APP/presenilin-1 (PS1) mice increased with age, indicating that miR-138 might decrease disintegrin and metalloproteinase 10 (ADAM10) expression and enhance Aβ synthesis in the mouse model. In addition, AD patients might have reduced levels of circHDAC9, which might boost miR-138 expression and reverse Sirt1 repression and excessive Aβ synthesis generated by miR-138 (Lu et al., 2019). UBE2A, the key effector of the ubiquitin-26 s proteasome system, which regulates the clearing of Aβ by proteolytic cleavage, is yet another mechanism that circRNAs might impact AD. However, it is reported that brains affected by sporadic AD have lower levels of UBE2A, which contributes to the accumulation of Aβ and the production of senile plaque deposits. In a study by Zhao and colleagues, the ciRS-7-miR-7-UBE2A circuit was considerably dysregulated in the neocortex and hippocampus CA1 of individuals with sporadic AD. The considerable reduction in UBE2A expression seemed to be driven by deficiencies in ciRS-7-mediated events, which caused overexpression of miR-7. Additionally, ciRS-7 decreased the proteins APP and BACE1 by enhancing their disintegration *via* the lysosome and proteasome (Zhao et al., 2016). Furthermore, a 2017 research by Shi and co-authors reported overexpression of the AD-associated sirtuin 7 gene inhibited the translation of the NF-κB while increasing the trafficking of the protein in the cytoplasm, where it promoted degradation of BACE1 and APP (Shi et al., 2017). Bigarré et al. (2021) reported that a rise in the expression levels of circ 0131235 in the middle temporal cortex of individuals with AD might be used as a biomarker for the disease. In addition, it is also hypothesized that enhanced circ 0131235 expressions could be part of a physiologic means to prevent Aβ aggregation (Bigarré et al., 2021).

### 2.2. Interplay between circRNAs, autophagy, and PD

PD, also known as paralysis agitans is the second most common progressive age-related motoric neurodegenerative disorder that affects approximately 1% of the population over the age of 65 years with a prevalence set to double by 2030 (Aarsland et al., 2021). Loss of dopaminergic neurons in the substantia nigra pars compacta results in slow and insidious cognitive decline along with disabling motor abnormalities including slow movements, tremor, poor balance and rigidity (Junn et al., 2009). It is suggested that circRNAs are important biomolecules for understanding and for addressing the initiation of PD neurodegenerative processes (Hanan et al., 2020). Studies have reported variable expression of circRNAs in the brains of patients with PD, and mounting evidence highlights their possible pathogenic role in PD development (Feng et al., 2020; Hanan et al., 2020; Kong et al., 2021). In one of the study, the authors reported alteration in the expression profiles of six circRNAs in the peripheral blood mononuclear cells obtained from the patients with PD compared with healthy control subjects including circ_0001566, circ_0006916, circ_0000497, circ_0001187, circ_0004368, and circ_0003848 (Ravanidis et al., 2021). One distinguishing feature of PD is the aberrant expression and aggregation of α-synuclein (SNCA), which is found in Lewy bodies. miR-7 has been reported to directly suppress SNCA expression by binding to the 3′ UTR of the SNCA gene and inhibiting its translation. The authors reported decreased expression of miR-7 in the substantia nigra Pars Compacta of patients with PD (McMillan et al., 2017). As circRNA ciRS-7 targets miR-7 (this circRNA contains 63 binding sites for miR-7), it probably serves as an important factor involved in the functioning of neurons as well as a responsible candidate in brain tumour development and neurological disorders (Hansen et al., 2013). Moreover, in another study done on PD, authors reported that clearance of SNCA and its aggregates were facilitated by miR-7 in autophagy dependent manner in differentiated ReNcell VM cells. The authors further revealed that miR-7 increases the formation of LC3 puncta and facilitated the conversion of LC3-I to LC3-II, which indicates increased autophagosome formation (Choi et al., 2018). In addition miRNAs (miR-7, miR-153, miR-214, and miR-133b) which targets SNCA have displayed protective role against the PD models (MPP+/MPTP). The authors revealed that by upregulating SNCA, miR-7 expression is involved in degeneration of the nigrostriatal system in the MPTP-induced neurotoxin model of PD in cultured cells and in mice (Junn et al., 2009). Moreover, miR-7 transfection resulted in more effective SNCA inhibition in the HeLa cell line in the absence of circRNA ciRS-7, showing that ciRS-7 may influence SNCA expression in miR-7-dependent manner, which is also correlated with the pathogenesis of PD (Wang et al., 2021b). In another study circSNCA was shown to sponge miR-7 and up-regulate the production of SNCA mRNA in SH-SY5Y cells, resulting in suppression of autophagy and upregulation of apoptosis (Sang et al., 2018). Authors reported that circSNCA down regulation increased the expression of the autophagy-related protein LC3B-II and the anti-apoptotic protein BCL2, demonstrating the suppression of apoptosis and the promotion of autophagy in PD. The researchers found that after treatment with a dopamine D2/D3 receptor agonist (pramipexole, commonly used in PD therapy), the expression profiles of SNCA and circSNCA were significantly reduced. The authors further reported that the expression of pro-apoptotic genes (CASP3, BAX, PTEN and P53) was reduced as a result of circSNCA downregulation (Sang et al., 2018).

One of the crucial mechanisms of circRNA in disease progression is through its interaction with disease-associated miRNAs (Ghosal et al., 2013). The role of circRNAs as a miRNA sponge in various diseases has also been investigated (Guo et al., 2014). In PD, circRNA zip-2 knockdown led to the reduction of SNCA protein aggregation by sponging miR-60, resulting in better survival outcomes of PD patients (Kumar et al., 2018). In another study authors reported that *via* sponging miR-7, circRNA s-7 promoted the expression of important genes associated with PD and AD (Lukiw, 2013). Suppressing circHIPK2 expression greatly reduced astrocyte activation through regulating autophagy and endoplasmic reticulum (ER) stress *via* targeting MIR124–2HG and SIGMAR (Huang et al., 2017a). CircDLGAP4 functions as a molecular sponge in PD patients and competes with MIR134-5p to suppress its activity. It encourages BECN1 and LC3-II expression, which increases autophagy, inhibits apoptosis, and decrease mitochondrial damage (Feng et al., 2020). In PD mouse models, circDLGAP4 is downregulated, which may contribute to the onset of PD by influencing cell viability, apoptosis, mitochondrial damage, and autophagy (Feng et al., 2020). It has been revealed that in PD, circSAMD4A altered the AMPK/mTOR cascade *via* miR-29c-3p, thus contributing to the death and autophagy of dopaminergic neurons. miR-124 has also been shown to protect dopaminergic neurons in PD *via* regulating the AMPK/mTOR mediated apoptosis and autophagy (Wang et al., 2021b).

In healthy brain’s substantia nigra, circRNAs accumulate in an age-dependent manner, whereas in the substantia nigra of patients with PD, the total number of circRNAs is reduced due to loss of this connection (Hanan et al., 2020). In one of the study, the authors reported increased expression of circSLC8A1 (this circRNA contains 7 binding sites for miR-128) in the substantia nigra of individuals with PD as well as in oxidative stress model created in cultured cells through Paraquat exposure. Authors further reported that expression profiles of miR-128 targets were also increased in PD individuals (Hanan et al., 2020). Above mentioned data has highlighted the importance of autophagy-associated circRNAs as potential therapeutic targets for PD. Studies of how circRNAs regulate autophagy during PD are still scarce and more investigations are needed.

### 2.3. Interplay between circRNAs, autophagy, and ALS

ALS is a neurodegenerative disease which damages upper and lower motor neurons of the brainstem, and spinal cord, causing paralysis of the voluntary muscles and the death of spinal motor neurons (Brown and Al-Chalabi, 2017; Ramesh and Pandey, 2017). There are number of factors that predispose ALS such as smoking, exposure to chemicals, metals and radiations leading to environmental causes of disease progression (Sutedja et al., 2009). Work done by Zarei et al. suggested that smoking and heavy metal cause mitochondrial damage, glutamate excitotoxicity as well as neurotoxicity (Zarei et al., 2015). Mutations in 40 different genes have been linked with the development of ALS including SOD1, C9ORF72, TARDBP, FUS, OPTN, and TANK-binding kinase 1 (TBK1; Cirulli et al., 2015). Furthermore, mutation in the autophagy pathway genes including p62/SQSTM, OPTN, TBK1, VCP, and C9ORF72 have been reported in ALS patients (Rudnick et al., 2017). The author showed that in both sporadic and familial ALS, mutations in autophagy gene were found. SOD1 was the first gene whose mutations were linked to ALS account accounting for about 3% of total ALS cases with more than 180 mutations discovered thus far linked to ALS (Peggion et al., 2022).

The accumulation of neurotoxic misfolded proteins, inclusions, and aggregates within motor neurons is the primary clinical signature in all cases of ALS. Autophagy has been found to be deregulated in both familial and sporadic cases of the disease (Vicencio et al., 2020). In order to protect the survival of extremely sensitive and specialized neurons, targeting autophagy through pharmacological autophagy-inducing agents has resulted in reduction in disease progression in different *in vitro* and *in vivo* models of neurodegenerative diseases (Amin et al., 2020). TBK1 cause the phosphorylation of autophagy adaptors which has a major role in both autophagy and mitophagy (Oakes et al., 2017). The authors showed that ALS causing gene have similar role in the autophagy such as SQSTM1/p62 and OPTN. Mutation in TBK1 lead to impairement of autophagy pathway that lead to accumulation of proteins and lead to pathogensis of ALS (Oakes et al., 2017). Another study by Kwiatkowski et al. revealed that mutation in FUS gene (which plays significant role in autophagy) lead to ALS (Kwiatkowski et al., 2009; Vance et al., 2009). Various atudies have reported shared mechanisms and connections between apparent dysregulations in autophagy and ALS pathophysiology (Nguyen et al., 2019).

CircRNAs are greatly localized in the tissues of human neurons, indicating that they may be significant in the control of the central nervous system (Li et al., 2019a). The study was conducted by Armakola et al. which revealed that deletion of Dbr1 gene which encodes an RNA lariat debranching enzyme was found to significantly lower cytoplasmic TAR DNA-binding protein 43 (TDP-43) toxicity. The main reason is that the lack of the debranching enzyme causes the development of intronic lariats (ciRNA), which bind to TDP-43 and reduce its toxicity (Armakola et al., 2012). Along with TDP-43, a mutation in the RBP known as fused in sarcoma (FUS) is also strongly linked to ALS. Proteins move from the nucleus to the cytoplasm as a result of FUS mutations, which also produce inclusion bodies in the cytoplasm and cause toxicity (Kwiatkowski et al., 2009, Vance et al., 2009). Additionally, FUS not only control the synthesis of circRNA and the introns that flank the back-splicing junctions in N2a cells through their binding (Errichelli et al., 2017). However, the formation of circRNAs (linked to the development of ALS) was shown to be reduced significantly by FUS depletion or its alteration (Verheijen and Pasterkamp, 2017). Another study by D’Amber et al. showed that there is no direct connection between mutant FUS and circ-Hdgfrp3 which is analyzed by post-acquisition and 3D rendering analyses, however circ-Hdgfrp3 is present in FUS-aggregates that connect circ-Hdgfrp3 with the p62/SQSTM1 protein (D'Ambra et al., 2021). These results suggests that it is possible that these granules will eventually be degraded *via* autophagy. The authors reproted that p62/SQSTM1 has connection with FUS inclusions in the brain and spinal cord of ALS patients (D'Ambra et al., 2021). Furthermore microarray profiling showed that hsa_circ_0023919, hsa_circ_0063411, and hsa_circ_0088036 have significant role in ALS patients (Dolinar et al., 2019). Evidences reported till now revealed that there is a correlation between circRNAs, autophagy and ALS which warrants further in-depth investigations.

### 2.4. Interplay between circRNAs, autophagy, and HD

**Figure 3:**
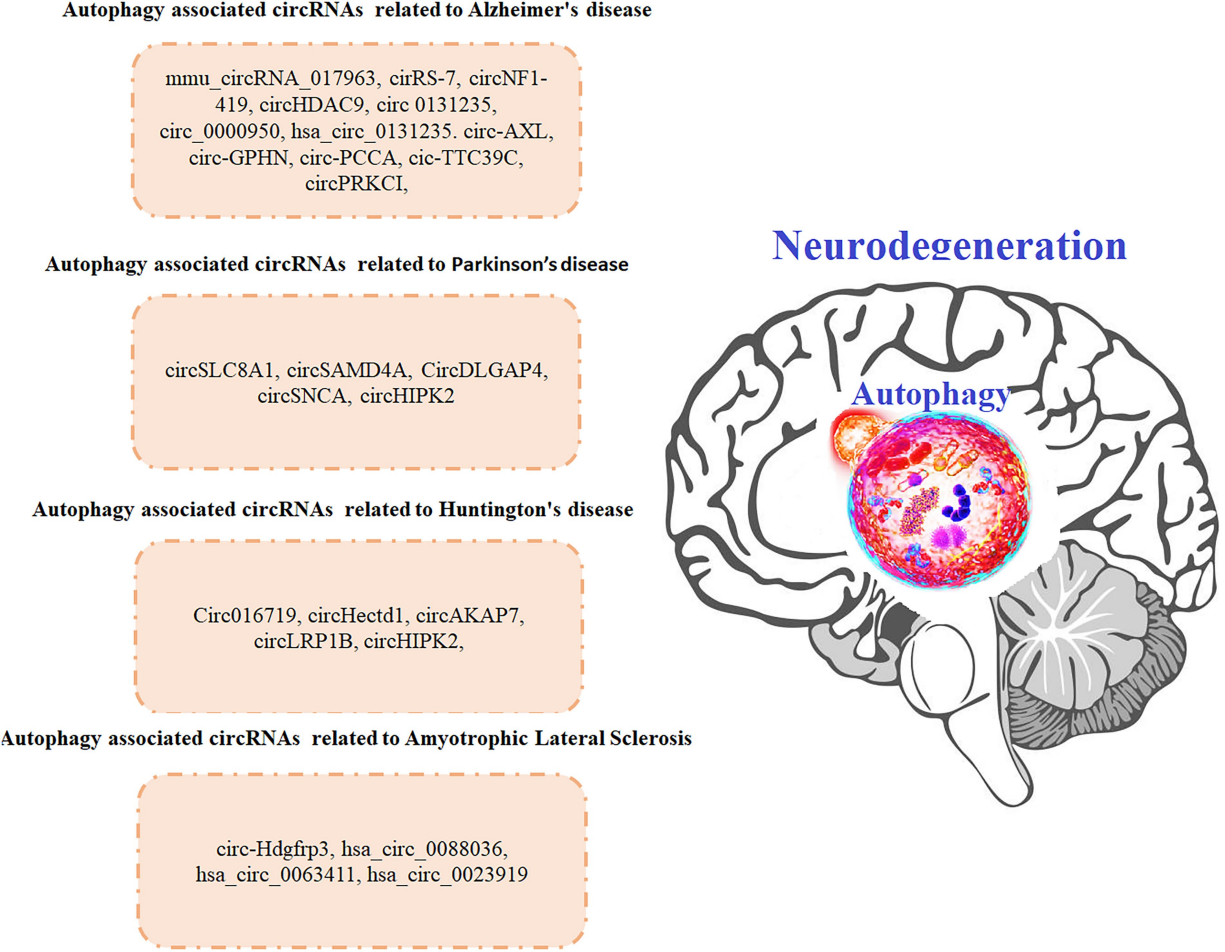
Autophagy associated circRNAs involved in neurodegenerative diseases.

Being a protein quality control system, autophagy process is involved in recycling obsolete cellular constituents as well as eliminating protein aggregates and organelles after being damaged. These substrates then reach lysosomes either by delivery within autophagosomes or endosomes. Disruptions caused by these interactions affect neuronal functions, which may cause several neurodegenerative diseases including HD. Studies have shown that autophagy disruption is linked with early cognitive changes in HD and is therefore a potential target for HD treatment (Grosso Jasutkar and Yamamoto, 2021). Furthermore, mutations of autophagy associated genes may specifically increase the risk of HD (Nixon, 2013), which is characterized by psychiatric disturbances cognitive dysfunction, and severe motor dysfunction (Sturrock and Leavitt, 2010). Autophagy associated miRNAs (a type of ncRNA and a target for circRNA) has been reported to be involved in the progression of HD (Choi and Cho, 2021). Expansion of the CAG repeat in the HTT gene is the main causative factor behind HD pathophysiology. In order to identify differentially expressed circRNAs in HD, Marin et al., did microarray analysis of murine cell line model expressing mutant HTT. The authors reported that differentially expressed circRNAs were found to regulate MAPK pathway, Long-term depression pathway as well as dopaminergic synapse which have been previously involved in HD physiopathology (Marfil-Marin et al., 2021). The authors concluded that in-depth understanding of the HD specific circRNA-miRNA-mRNA axis can lead to identify novel biomarkers and potential therapeutic targets for HD. HTT has been previously reported to play a crucial role in selective autophagy, where the loss of the polyQ stretch enhances autophagic capacity in neurons (Zheng et al., 2010). Moreover HTT gene has also found to be structurally similar to selective autophagy proteins known as Atg11, Atg23, and Vac8 (Steffan, 2010). It also promotes autophagy initiation, where as its loss in CNS has been suggested to cause protein accumulation, which might contribute to HD pathogenesis (Gelman et al., 2015). Researchers are still working to find out possible associations and links between autophagic processes and HD pathogenesis (Croce and Yamamoto, 2019). In one of the study, the author identified a circRNA derived from HTT locus in brain districts and in iPS-derived neuronal cell lines. The authors reported that overexpression of the circHTT might cause alteration in the wild-type and mutant HTT expression (Döring et al., 2021). The authors concluded that circRNAs might serve as innovative avenues for therapeutic intervention to treat HD. A number of studies have explored the relationship between neurological disorders, autophagy and circRNAs but association of circRNAs and autophagy with HD is still in infancy and therefore need further studies. In one of the study the authors reported that upregulation of circ016719 expression (which binds miR-29c) in neurons promotes autophagy. Circ016719 ultimately increased the expression of MAP2K6, which then promotes the expression of BECN1, thereby increasing the rate of autophagy in neurons. The authors further reported that circ_016719 knockdown significantly inhibited autophagy (Tang et al., 2020). In another study miR-29c was found among nine miRNAs (miR-128, miR-132, miR-22, miR-218, miR-222, miR-29c, miR-138, miR-344, and miR-674) which were downregulated in YAC128 transgenic mice expressing a full-length mutant HTT (Dong and Cong, 2021). Above mentioned studies supports the notion that circ016719/miR-29c/autophagy axis should be explored in HD models. In another study, upregulation of circHectd1 in astrocytes was found to promote the expression of TIPARP (through miR-142-TIPARP axis), which then induces the expression of LC3-II, thereby activating autophagy (Han et al., 2018). miR-142 has been reported to be linked with HD (Martí et al., 2010) which shows that circHectd1/miR-142/autophagy axis might have implications in HD. In another study circAKAP7 was shown to target miR-155-5p to promote the expression of ATG12, thereby increasing the risk of autophagy (Xu et al., 2020). One of the study reported that in the animal models, overexpression of miR-155 lowered the mutant HTT aggregates in cortex and striatum, as result there was improved performance in behavioral tests (Martinez and Peplow, 2021). A number of other circRNAs might contribute to HD *via* regulating autophagy such as circLRP1B that inhibits miR-27a-3p, circDLGAP4 that inhibits miR-134-5p, circHIPK2 that inhibits miR-124. We believe that by regulating autophagy, circRNAs might impact the development and/or recovery of HD. However, this hypothesis needs to be confirmed by studying the role of autophagy associated circRNAs in HD development (Wang et al., 2022; Figure 3).

### 2.5. Interplay between circRNAs, autophagy and SMA

SMA is a devastating genetic neuromuscular condition and the primary cause of infant death, which weakens the muscles resulting in low amounts of the Survival Motor Neuron (SMN) protein (Sansa et al., 2021). There are five types of SMA: Types 0, 1, 2, 3, and 4. SMA 1 (also called Werdnig-Hoffmann disease) is the most common form of the condition, which is deadly within the initial 2 years of life (Park et al., 2010). SMA can appear in a variety of severity levels. Patients with lower spinal cord-motor neuron impairment cause increasing muscle weakness. The SMN protein was given the name “survival motor neuron” because of the apparent importance of protein to the motor system and the finding that the knockout of SMN in mice was embryonically lethal (Schrank et al., 1997). Recent studies have shown that SMN genes generates a huge repertoire of circRNAs through inter-intronic secondary structures (Luo et al., 2022; Singh et al., 2022). There are four subtypes of SMN circRNAs namely Type 1, 2, 3 and 4 circRNAs (Ottesen and Singh, 2020). Most of the SMA data has been generated using SMA mouse models which have confirmed the significant function of autophagy during SMA. In particular, SMA is caused through oxidative stress which increases expression of a mutated superoxide dismutase protein (SODG93A) by triggering autophagy (Dobrowolny et al., 2008). Studies have shown that ncRNAs including circRNAs could be potentially exploited for developing additional SMA therapies (Singh et al., 2020). Research has indicated that autophagy is disrupted during the initial regulatory stages in SMA. The role of circRNAs in SMA pathogenesis is still in initial phases.

miRNAs which are the targets of circRNAs have been exploited in SMA and reports have revealed their neuroprotective abilities (Gandhi et al., 2021). A number of miRNAs including miR-23a (reduced the motor neuron death, increased the motor neuron size as well as muscle fiber area), miR-375 (protects the neuron from apoptosis by inhibiting the tumour suppressor gene p53), miR-9 (crucial modulator associated with SMA severity), miR-146a (regulates crucial signaling pathways in motor neurons), miR-183 (increases the motor neurons survival and improves the motor function of mice with mutant SMN), miR-206 (role in survival endogenous mechanism), and miR-431 (rescue the motor neuron neurite length phenotype through targeting chondrolectin) have been considered as potential therapeutic ncRNAs for treating SMA (Gandhi et al., 2021). These miRNAs have been implicated in autophagy as well as targets of circRNAs in separate studies which warrants further investigations of circRNAs/miRNAs/autophagy axis in SMA (Chang et al., 2012; Zhang et al., 2015; Si et al., 2018; Huang et al., 2019; Shang et al., 2021; Wei et al., 2021).

### 2.6. Interplay between circRNAs, autophagy, and FRDA

FRDA, a multi-system, autosomal recessive neurodegenerative disorder and is the most abundantly existing type of hereditary ataxia (Keita et al., 2022). This illness usually starts before the age of 25 with subsequent disease progression including dorsal root ganglia, sensory peripheral nerves, corticospinal tracts, and dentate nuclei of the cerebellum (Santos et al., 2010). Apart from these indications, a very high percentage of patients also advance to hypertrophic cardiomyopathy leading to lower life expectancy. FRDA is caused by the loss of function mutations in FXN gene which is responsible for causing FRDA. FXN gene (9q13-21) actually encodes for Frataxin protein, that is a small protein (210-amino-acids in length) produced as a precursor polypeptide and later exported to mitochondria where proteolytic cleavage occurs to produce the mature type called as m-FXN (Campuzano et al., 1996; Koutnikova et al., 1997; Priller et al., 1997; Schmucker et al., 2008). It is mainly initiated by a homozygous GAA repeat expansion mutation within the intron 1 of the FXN gene (Campuzano et al., 1996). Some of patients are also heterozygous, having one pathogenic mutation (insertion, deletion, point mutation) in one of the FXN allele while having the GAA repeat expansion on the other making them compound heterozygous (Santos et al., 2010). Such expansion in FXN genes develop repeat-dependent epigenetic silencing signals, including repressive histone marks and DNA hypermethylation, that are mostly present in intronic region (Greene et al., 2007). This results in almost 30% reduced production of protein frataxin in the body, consequently, reduced import into mitochondria and reduced mitochondrial functioning, leading to subsequent oxidative stress and cell death.

Studies have reported that autophagy has a crucial role in all neurodegenerative diseases including FRDA. Many experiments have been performed on various cell lines and models to decipher the exact role and mechanism of FRDA and the role of autophagy in its pathogenesis. It is yet to be clarified whether increase in FRDA associated autophagy is a neurodegenerative mechanism (Simon et al., 2004) or it may serve a protective role against stress (Bolinches-Amoros et al., 2014). In one set of experiments, Caenorhabditis elegans (C. elegans) was used as a model organism revealing that a complete knock-out of the frataxin ortholog (frh-1) or severe insufficiency of additional nuclear-encoded mitochondrial respiratory chain (MRC) proteins results in pathological phenotypes such as shorter lifespan and arrested development (Rea et al., 2007). Inversely, partial suppression of this gene, as well as frh-1, results in activation of some beneficial adaptive responses leading to life span extension (Torgovnick et al., 2010). It is also observed that FXN protein expression must be considerably reduced to activate autophagy, which results in reducing lipid content and extended lifespan and in C. elegans. There might exist a causal connection among initiation of autophagy and extended lifespan subsequent to reduced FXN protein expression. This might provide a rationale to further investigate autophagy in the pathogenesis and treatment of FRDA.

In one of the experiment, the authors used gene silencing in human neuroblastoma cell line, SH-SY5Y, to reveal effects on cellular and mitochondrial functioning due to FXN protein deficiency (Bolinches-Amoros et al., 2014). Neuroblastoma is actually a tumor made from the neural crest, like DRG neurons that’s why neuroblastoma cell line seems a better cellular model to study ataxia and other related disorders. The authors observed that the silencing of FXN gene caused mitochondrial dysfunction leading to cellular senescence and an increase in autophagy. However, the authors argue that the induced autophagy might play a protective role in FRDA. Actually neurons are post mitotic cells whose survival is dependent upon autophagy (Lee, 2009). On the other hand, the senescence phenotype observed can be linked to degeneration observed in the FRDA patients. Collectively, possibility exists that autophagy is initiated to protect FXN-deficient DRG neurons from stress, but then again observed degeneration might be because of induction of additional pathways in response to various stresses such as oxidative stress. The discovery of the Drosophila FXN ortholog, fh, (Cañizares et al., 2000) led to the development of fly models of FRDA that can be used to explore FXN function (Calap-Quintana et al., 2015). Experiments conducted using Drosophila model of FRDA, suggested that autophagy is undeniably essential for the shielding effect of rapamycin in hyperoxia (Calap-Quintana et al., 2015).

There are several theories proposed, on the basis of growing evidence, of circRNA role in neurological diseases. Firstly, it is postulated that all RNA molecules, both non-coding as well as protein-coding RNAs communicate with and co-regulate each other by competing for binding to shared miRNAs (Salmena et al., 2011). This hypothesis collectively refers to all RNAs as ceRNA (competitive endogenous RNAs; Salmena et al., 2011). Importantly, there is mounting data that suggests that the RNA communication system (ceRNA) is disrupted in these diseases resulting in a pathogenic state. With reference to FRDA, we can observe that the nuclear production of precursor FXN protein to its mature delivery to mitochondria, highly depends on such interconnected regulation among ceRNAs including circRNAs. Secondly, it has been also suggested that such communication between RNA molecules may play an important role in several repeat-associated diseases including FRDA. FRDA, as already discussed, is mainly caused by homozygous GAA repeat expansion mutations in intron 1. There is a strong argument in Myotonic dystrophy type 1 disease, which is also caused by CTG repeats in the 3′ untranslated region of the dystrophia myotonica protein kinase (DMPK) gene (Brook et al., 1992). The expression of mutation-containing transcripts causes the sequestration of muscle blind-like (MBNL) proteins which normally regulate alternative splicing of pre-messenger RNAs (pre-mRNAs) encoding proteins critical for skeletal, cardiac, and nervous system function (Miller et al., 2000; Pascual et al., 2006). Thus, their sequestration and functional inadequacy results in an aberrant alternative splicing of many target genes. One of the study suggested a role of MBNLs in the production of circRNAs (Ashwal-Fluss et al., 2014). The production of circRNAs might ensue both post-transcriptionally and co-transcriptionally (Wilusz and Sharp, 2013; Ashwal-Fluss et al., 2014; Kramer et al., 2015). Also, their biogenesis competes with the production of linear transcripts (mRNA). Apart from this phenomenon, two other important roles have been proposed of circRNAs which might play a crucial role in pathogenesis of FRDA including contribution in protein and/or RNA transport (Memczak et al., 2013) and regulation of the synaptic functions in neural tissue (You et al., 2015).

There are several studies which have deciphered the role of miRNAs in FRDA. CircRNAs act as sponges for these miRNAs extending their role in the pathogenesis by effecting the key molecules involved in the autophagy associated pathways thereby regulating the biological functions of these cells. For example, those miRNAs targeting mTOR and AMPK pathway might play a crucial role in the progression of FRDA. One such example is hsa-miR223-3p, which is slightly upregulated in FRDA patients (Seco-Cervera et al., 2017; Quatrana et al., 2022). This miRNA is known to be inversely associated with HCLS1 associated protein X-1 (HAX-1). It has been found that Hax-1 plays an important role in the development of the central nervous system and in the pathophysiology of some neurological diseases. The anti-apoptotic protein HAX-1 has been proposed to modulate mitochondrial membrane potential, calcium signaling and actin remodeling (Dong et al., 2022). It is possible that this miRNA might negatively regulate HAX-1 protein thus reducing the anti-apoptotic effect and in turn promotes autophagy in FRDA patients. Another miRNA hsa-miR-886-3p (miR-886-3p) was observed to be upregulated in FRDA patient’s cells as well as peripheral patient’s blood samples (Dantham et al., 2018). The study identified significant deregulation of following miRNAs; hsa-miR-15a-5p, hsa-miR-26a-5p, hsa-miR-29a-3p, hsa-miR-223–3p, hsa-24–3p, and hsa-miR-21–5p which are shown to be associated with neurodegenerative and other clinical features in FRDA (Dantham et al., 2018). These miRNAs have been implicated in autophagy as well as targets of circRNAs in separate studies which warrants further investigations of circRNAs/miRNAs/autophagy axis in FRDA. However, further studies are warranted to find the exact mechanism of autophagy associated circRNAs and their role in FRDA.

## 3. Conclusion

CircRNAs tends to accumulate in the aging central nervous system which makes them potentially suitable therapeutic targets as well as diagnostic and prognostic biomarkers for age-related diseases, including neurodegenerative diseases. Autophagy failure and autophagosomal dysfunction have been implicated in the pathogenesis of several neurodegenerative diseases, as a result, it has gained increasing attention and interest in neuronal cell biology. Research has shown that by influencing autophagy, circRNAs indirectly regulate cell proliferation, which plays an important role in the development of neurodegenerative diseases. Despite mounting research on circRNAs and their connection with autophagy, the mechanism underlying circRNA-mediated regulation of autophagy in the pathogenesis of neurodegeneration remains to be explored. There are many questions that still needs to be addressed regarding complex regulatory roles of autophagy associated circRNAs in brain. Studies evaluating the regulatory roles of circRNAs in neurodegenerative diseases are still limited, however reports suggested that circRNA play regulatory roles through diverse mechanisms, including transcription and splicing regulation, as ceRNA *via* sponging miRNAs, mRNA traps, post-translational modifications as well as through epigenetic modification. Some of the regulatory roles of circRNAs in neurodegenerative diseases have been discussed in this manuscript (e.g., circHectd1-miR-142-TIPARP axis, ciRS-7-miR-7/UBE2A axis, circGLIS3/miR-203 axis, circSNCA/miR-7/SNCA axis, circzip-2/miR-60/SNCA axis, circHIPK2/miR124/2HG axis, circHIPK2/miR124/SIGMAR axis, circSAMD4A/miR-29c-3p AMPK/mTOR axis, circ016719/miR-29c/MAP2K6 axis, and circAKAP7/miR-155-5p/ATG12 axis etc.). The regulatory mechanisms of autophagy associated circRNA in neurodegenerative diseases are remarkably complex. It is therefore essential to use mammalian disease models and to employ state of the art genetic and molecular tools to decipher the role of autophagy associated circRNAs under normal and pathological conditions. Such studies will likely increase our understanding of the complex regulatory roles of autophagy associated circRNAs and will also uncover new therapeutic opportunities.

## 4. Future perspectives

With the advancement and progress in circRNA research, the mechanism by which autophagy associated circRNAs influence neurodegenerative diseases is gradually being discovered. Before they can be considered for clinical implications, a number of major questions should be addressed, e.g., what role do autophagy associated circRNAs play during different stages of neurodegeneration, their effects on neuroplasticity, neurogenesis, and behavior and how circRNAs influence multiple signaling pathways at the synapse. Moreover in order to determine how autophagy associated circRNAs impact neurodegeneration, transgenic animal models, cell lines as well as efficient and conditional knockdowns, or over-expression models of specific circRNAs should be developed to establish suitable *in-vivo* and *in-vitro* systems. Mapping region-specific autophagy associated circRNAs across the entire brain would also be useful. In the coming years, studies that expand our knowledge on in-depth mechanistic understanding of autophagy associated circRNAs will facilitate the development of specific and effective approaches to target these circRNAs *in vivo* will be the key in advancing the clinical potential of circRNA-based therapeutics. Furthermore, research efforts need to be channeled toward dissecting and characterizing their specific regulatory roles and evaluating the extent to which they contribute towards pathogenesis of neurodegenerative diseases.

## Author contributions

FA and BY: conceptualization and review and editing. RB, UA, AO, AN, AI, SuK, IH, SaK, and MA: data collection and literature review. FA and RB: writing original draft. All authors contributed to the article and approved the submitted version.

## Conflict of interest

The authors declare that the research was conducted in the absence of any commercial or financial relationships that could be construed as a potential conflict of interest.

The handling editor JW declared a shared parent affiliation with the author BY at the time of review.

## Publisher’s note

All claims expressed in this article are solely those of the authors and do not necessarily represent those of their affiliated organizations, or those of the publisher, the editors and the reviewers. Any product that may be evaluated in this article, or claim that may be made by its manufacturer, is not guaranteed or endorsed by the publisher.

## References

[ref1] Aarsland D. Batzu L. Halliday G. M. Geurtsen G. J. Ballard C. Ray Chaudhuri K.. (2021). Parkinson disease-associated cognitive impairment. Nat. Rev. Dis. Primers 7, 1–21. doi: 10.1038/s41572-021-00280-3 34210995

[ref2] Alcendor J. (2020). Interactions between amyloid-β proteins and human brain pericytes: implications for the pathobiology of Alzheimer’s disease. J. Clin. Med. 9:1490. doi: 10.3390/jcm9051490, PMID: 32429102 PMC7290583

[ref3] Amin A. Perera N. D. Beart P. M. Turner B. J. Shabanpoor F. (2020). Amyotrophic lateral sclerosis and autophagy: dysfunction and therapeutic targeting. Cells 9:2413. doi: 10.3390/cells9112413, PMID: 33158177 PMC7694295

[ref4] Armakola M. Higgins M. J. Figley M. D. Barmada S. J. Scarborough E. A. Diaz Z.. (2012). Inhibition of RNA lariat debranching enzyme suppresses TDP-43 toxicity in ALS disease models. Nat. Genet. 44, 1302–1309. doi: 10.1038/ng.2434, PMID: 23104007 PMC3510335

[ref5] Ashwal-Fluss R. Meyer M. Pamudurti N. R. Ivanov A. Bartok O. Hanan M.. (2014). circRNA biogenesis competes with pre-mRNA splicing. Mol. Cell 56, 55–66. doi: 10.1016/j.molcel.2014.08.019, PMID: 25242144

[ref6] Association, A. S (2010). 2010 Alzheimer's disease facts and figures. Alzheimers Dement. 6, 158–194. doi: 10.1016/j.jalz.2010.01.009, PMID: 20298981

[ref7] Association, A. S (2019). 2019 Alzheimer's disease facts and figures. Alzheimers Dement. 15, 321–387. doi: 10.1016/j.jalz.2019.01.010

[ref8] Bai Y. Zhang Y. Hua J. Yang X. Zhang X. Duan M.. (2016). Silencing microRNA-143 protects the integrity of the blood-brain barrier: implications for methamphetamine abuse. Sci. Rep. 6, 1–15. doi: 10.1038/srep35642 27767041 PMC5073292

[ref9] Bateman R. J. Munsell L. Y. Morris J. C. Swarm R. Yarasheski K. E. Holtzman D. M. (2006). Human amyloid-β synthesis and clearance rates as measured in cerebrospinal fluid in vivo. Nat. Med. 12, 856–861. doi: 10.1038/nm1438, PMID: 16799555 PMC2983090

[ref10] Bigarré I. M. Trombetta B. A. Guo Y. J. Arnold S. E. Carlyle B. C. (2021). IGF2R circular RNA hsa_circ_0131235 expression in the middle temporal cortex is associated with AD pathology. Brain Behav. 11:e02048. doi: 10.1002/brb3.2048, PMID: 33704916 PMC8035435

[ref11] Bolinches-Amoros A. Mollá B. Pla-Martin D. Palau F. Gonzalez-Cabo P. (2014). Mitochondrial dysfunction induced by frataxin deficiency is associated with cellular senescence and abnormal calcium metabolism. Front. Cell. Neurosci. 8:124. doi: 10.3389/fncel.2014.00124, PMID: 24860428 PMC4026758

[ref12] Brook J. D. Mccurrach M. E. Harley H. G. Buckler A. J. Church D. Aburatani H.. (1992). Molecular basis of myotonic dystrophy: expansion of a trinucleotide (CTG) repeat at the 3′ end of a transcript encoding a protein kinase family member. Cells 68, 799–808. doi: 10.1016/0092-8674(92)90154-5, PMID: 1310900

[ref13] Brown R. H. Al-Chalabi A. (2017). Amyotrophic lateral sclerosis. N. Engl. J. Med. 377, 162–172. doi: 10.1056/NEJMra1603471, PMID: 28700839

[ref14] Burgaletto C. Platania C. B. M. Di Benedetto G. Munafò A. Giurdanella G. Federico C.. (2021). Targeting the miRNA-155/TNFSF10 network restrains inflammatory response in the retina in a mouse model of Alzheimer’s disease. Cell Death Dis. 12, 1–15. doi: 10.1038/s41419-021-04165-x 34611142 PMC8492692

[ref15] Cai H. Li Y. Niringiyumukiza J. D. Su P. Xiang W. (2019). Circular RNA involvement in aging: an emerging player with great potential. Mech. Ageing Dev. 178, 16–24. doi: 10.1016/j.mad.2018.11.002, PMID: 30513309

[ref16] Calap-Quintana P. Soriano S. Llorens J. V. Al-Ramahi I. Botas J. Moltó M. D.. (2015). TORC1 inhibition by rapamycin promotes antioxidant defences in a drosophila model of Friedreich’s ataxia. PLoS One 10:e0132376. doi: 10.1371/journal.pone.0132376, PMID: 26158631 PMC4497667

[ref17] Campuzano V. Montermini L. Molto M. D. Pianese L. Cossée M. Cavalcanti F.. (1996). Friedreich's ataxia: autosomal recessive disease caused by an intronic GAA triplet repeat expansion. Science 271, 1423–1427. doi: 10.1126/science.271.5254.1423, PMID: 8596916

[ref18] Cañizares J. N. Blanca J. M. Navarro J. A. Monrós E. Palau F. Moltó M. A. D. (2000). dfh is a drosophila homolog of the Friedreich's ataxia disease gene. Gene 256, 35–42. doi: 10.1016/S0378-1119(00)00343-7, PMID: 11054533

[ref19] Cao D. Zhu H. Zhao Q. Huang J. Zhou C. He J.. (2020). MiR-128 suppresses metastatic capacity by targeting metadherin in breast cancer cells. Biol. Res. 53, 1–13. doi: 10.1186/s40659-020-00311-5 32993809 PMC7526227

[ref20] Cardoso A. L. Guedes J. R. Almeida L P. D. E. Pedroso De Lima M. C. (2012). miR-155 modulates microglia-mediated immune response by down-regulating SOCS-1 and promoting cytokine and nitric oxide production. Immunology 135, 73–88. doi: 10.1111/j.1365-2567.2011.03514.x, PMID: 22043967 PMC3246654

[ref21] Chandran A. Rochet J.-C. (2022). Shining a light on autophagy in neurodegenerative diseases. J. Biol. Chem. 298:101437. doi: 10.1016/j.jbc.2021.101437, PMID: 34801556 PMC8718947

[ref22] Chang Y. Yan W. He X. Zhang L. Li C. Huang H.. (2012). miR-375 inhibits autophagy and reduces viability of hepatocellular carcinoma cells under hypoxic conditions. Gastroenterology 143:e8. doi: 10.1053/j.gastro.2012.04.009 22504094

[ref23] Chen H.-H. Eteleeb A. Wang C. Fernandez M. V. Budde J. P. Bergmann K.. (2022). Circular RNA detection identifies circPSEN1 alterations in brain specific to autosomal dominant Alzheimer's disease. Acta Neuropathol. Commun. 10, 1–11. doi: 10.1186/s40478-022-01328-5 35246267 PMC8895634

[ref24] Cheng Q. Cao X. Xue L. Xia L. Xu Y. (2019). CircPRKCI-miR-545/589-E2F7 axis dysregulation mediates hydrogen peroxide-induced neuronal cell injury. Biochem. Biophys. Res. Commun. 514, 428–435. doi: 10.1016/j.bbrc.2019.04.131, PMID: 31053300

[ref25] Choi S. H. Cho K. (2021). LAMP2A-mediated autophagy involved in Huntington’s disease progression. Biochem. Biophys. Res. Commun. 534, 561–567. doi: 10.1016/j.bbrc.2020.11.042, PMID: 33239172

[ref26] Choi D. C. Yoo M. Kabaria S. Junn E. (2018). MicroRNA-7 facilitates the degradation of alpha-synuclein and its aggregates by promoting autophagy. Neurosci. Lett. 678, 118–123. doi: 10.1016/j.neulet.2018.05.009, PMID: 29738845 PMC5990033

[ref27] Cirulli E. T. Lasseigne B. N. Petrovski S. Sapp P. C. Dion P. A. Leblond C. S.. (2015). Exome sequencing in amyotrophic lateral sclerosis identifies risk genes and pathways. Science 347, 1436–1441. doi: 10.1126/science.aaa3650, PMID: 25700176 PMC4437632

[ref28] Croce K. R. Yamamoto A. (2019). A role for autophagy in Huntington's disease. Neurobiol. Dis. 122, 16–22. doi: 10.1016/j.nbd.2018.08.010, PMID: 30149183 PMC6364695

[ref29] Crous-Bou M. Minguillón C. Gramunt N. Molinuevo J. L. (2017). Alzheimer’s disease prevention: from risk factors to early intervention. Alzheimers Res. Ther. 9, 1–9. doi: 10.1186/s13195-017-0297-z 28899416 PMC5596480

[ref30] D’anca M. Buccellato F. R. Fenoglio C. Galimberti D. (2022). Circular RNAs: emblematic players of neurogenesis and neurodegeneration. Int. J. Mol. Sci. 23:4134. doi: 10.3390/ijms23084134, PMID: 35456950 PMC9032451

[ref31] D'ambra E. Santini T. Vitiello E. Duva S. Silenzi V. Morlando M.. (2021). Circ-Hdgfrp3 shuttles along neurites and is trapped in aggregates formed by ALS-associated mutant FUS. iScience 24:103504. doi: 10.1016/j.isci.2021.103504, PMID: 34934923 PMC8661529

[ref32] Dantham S. Srivastava A. K. Gulati S. Rajeswari M. R. (2018). Differentially regulated cell-free microRNAs in the plasma of friedreich's ataxia patients and their association with disease pathology. Neuropediatrics 49, 035–043. doi: 10.1055/s-0037-1607279, PMID: 29179232

[ref33] Delavar M. R. Baghi M. Safaeinejad Z. Kiani-Esfahani A. Ghaedi K. Nasr-Esfahani M. H. (2018). Differential expression of miR-34a, miR-141, and miR-9 in MPP+-treated differentiated PC12 cells as a model of Parkinson's disease. Gene 662, 54–65. doi: 10.1016/j.gene.2018.04.010, PMID: 29631008

[ref34] Diling C. Yinrui G. Longkai Q. Xiaocui T. Yadi L. Xin Y.. (2019). Circular RNA NF1-419 enhances autophagy to ameliorate senile dementia by binding Dynamin-1 and adaptor protein 2 B1 in AD-like mice. Aging (Albany NY) 11, 12002–12031. doi: 10.18632/aging.102529, PMID: 31860870 PMC6949063

[ref35] Dobrowolny G. Aucello M. Rizzuto E. Beccafico S. Mammucari C. Bonconpagni S.. (2008). Skeletal muscle is a primary target of SOD1G93A-mediated toxicity. Cell Metab. 8, 425–436. doi: 10.1016/j.cmet.2008.09.002, PMID: 19046573

[ref36] Dolinar A. Koritnik B. Glavač D. Ravnik-Glavač M. (2019). Circular RNAs as potential blood biomarkers in amyotrophic lateral sclerosis. Mol. Neurobiol. 56, 8052–8062. doi: 10.1007/s12035-019-1627-x, PMID: 31175544 PMC6834740

[ref37] Dong X. Cong S. (2021). MicroRNAs in Huntington’s disease: diagnostic biomarkers or therapeutic agents? Front. Cell. Neurosci. 313. doi: 10.3389/fncel.2021.705348 PMC8377808 34421543

[ref38] Dong Q. Li D. Zhao H. Zhang X. Liu Y. Hu Y.. (2022). Anti-apoptotic HAX-1 suppresses cell apoptosis by promoting c-Abl kinase-involved ROS clearance. Cell Death Dis. 13, 1–14. doi: 10.1038/s41419-022-04748-2 PMC8979985 35379774

[ref39] Döring J. Ferrari S. Monziani A. Pegorar C. O. Tripathi T. Di Leva F.. (2021). A04 Circhtt, a circular rna from the huntington’s disease gene locus: Functional characterization and possible implications for disease modulation BMJ Publishing Group Ltd.

[ref40] Dube U. Del-Aguila J. L. Li Z. Budde J. P. Jiang S. Hsu S.. (2019). An atlas of cortical circular RNA expression in Alzheimer disease brains demonstrates clinical and pathological associations. Nat. Neurosci. 22, 1903–1912. doi: 10.1038/s41593-019-0501-5, PMID: 31591557 PMC6858549

[ref41] Egorova N. Dhollander T. Khlif M. S. Khan W. Werden E. Brodtmann A. (2020). Pervasive white matter fiber degeneration in ischemic stroke. Stroke 51, 1507–1513. doi: 10.1161/STROKEAHA.119.028143, PMID: 32295506

[ref42] Errichelli L. Dini Modigliani S. Laneve P. Colantoni A. Legnini I. Capauto D.. (2017). FUS affects circular RNA expression in murine embryonic stem cell-derived motor neurons. Nat. Commun. 8, 1–11. doi: 10.1038/ncomms14741 28358055 PMC5379105

[ref43] Fan W. Wen X. Xiao H. Yang Q. Liang Z. (2018). MicroRNA-29a enhances autophagy in podocytes as a protective mechanism against high glucose-induced apoptosis by targeting heme oxygenase-1. Eur. Rev. Med. Pharmacol. Sci. 22, 8909–8917. doi: 10.26355/eurrev_201812_16660, PMID: 30575934

[ref44] Feng X. Hu J. Zhan F. Luo D. Hua F. Xu G. (2021). MicroRNA-138-5p regulates hippocampal neuroinflammation and cognitive impairment by NLRP3/caspase-1 signaling pathway in rats. J. Inflamm. Res. 14, 1125–1143. doi: 10.2147/JIR.S304461, PMID: 33814920 PMC8009546

[ref45] Feng Z. Zhang L. Wang S. Hong Q. (2020). Circular RNA circDLGAP4 exerts neuroprotective effects via modulating miR-134-5p/CREB pathway in Parkinson’s disease. Biochem. Biophys. Res. Commun. 522, 388–394. doi: 10.1016/j.bbrc.2019.11.102, PMID: 31761328

[ref46] Filippone A. Esposito E. Mannino D. Lyssenko N. Praticò D. (2022). The contribution of altered neuronal autophagy to neurodegeneration. Pharmacol. Ther. 108178. doi: 10.1016/j.pharmthera.2022.108178 PMC9510148 35351465

[ref47] Fletcher K. Klosterman S. J. Derevnina L. Martin F. Bertier L. D. Koike S.. (2018). Comparative genomics of downy mildews reveals potential adaptations to biotrophy. BMC Genomics 19, 1–23. doi: 10.1186/s12864-018-5214-8 30486780 PMC6264045

[ref48] Fu J. Peng L. Tao T. Chen Y. Li Z. Li J. (2019). Regulatory roles of the miR-200 family in neurodegenerative diseases. Biomed. Pharmacother. 119:109409. doi: 10.1016/j.biopha.2019.109409, PMID: 31518873

[ref49] Gan X. Zhu H. Jiang X. Obiegbusi S. C. Yong M. Long X.. (2020). CircMUC16 promotes autophagy of epithelial ovarian cancer via interaction with ATG13 and miR-199a. Mol. Cancer 19, 1–13. doi: 10.1186/s12943-020-01163-z 32111227 PMC7047414

[ref50] Gandhi G. Abdullah S. Foead A. I. Yeo W. W. Y. (2021). The potential role of miRNA therapies in spinal muscle atrophy. J. Neurol. Sci. 427:117485. doi: 10.1016/j.jns.2021.117485, PMID: 34015517

[ref51] Gandhi S. Abramov A. Y. (2012). Mechanism of oxidative stress in neurodegeneration. Oxidative medicine and cellular longevity, 2022. 10.1155/2012/428010 PMC3362933 22685618

[ref52] Gelman A. Rawet-Slobodkin M. Elazar Z. (2015). Huntingtin facilitates selective autophagy. Nat. Cell Biol. 17, 214–215. doi: 10.1038/ncb3125, PMID: 25720962

[ref53] Ghosal S. Das S. Sen R. Basak P. Chakrabarti J. (2013). Circ2Traits: a comprehensive database for circular RNA potentially associated with disease and traits. Front. Genet. 4:283. doi: 10.3389/fgene.2013.00283, PMID: 24339831 PMC3857533

[ref54] Greene E. Mahishi L. Entezam A. Kumari D. Usdin K. (2007). Repeat-induced epigenetic changes in intron 1 of the frataxin gene and its consequences in Friedreich ataxia. Nucleic Acids Res. 35, 3383–3390. doi: 10.1093/nar/gkm271, PMID: 17478498 PMC1904289

[ref55] Grosso Jasutkar H. Yamamoto A. (2021). Do changes in synaptic autophagy underlie the cognitive impairments in Huntington’s disease? J. Huntington's Dis. 10, 227–238. doi: 10.3233/JHD-200466, PMID: 33780373 PMC8293641

[ref56] Gruner H. Cortes-Lopez M. Cooper D. Bauer M. Miura P. (2016). CircRNA accumulation in the aging mouse brain. Sci. Rep. 6:38907. doi: 10.1038/srep38907, PMID: 27958329 PMC5153657

[ref57] Gu D.-N. Jiang M.-J. Mei Z. Dai J.-J. Dai C.-Y. Fang C.. (2017). microRNA-7 impairs autophagy-derived pools of glucose to suppress pancreatic cancer progression. Cancer Lett. 400, 69–78. doi: 10.1016/j.canlet.2017.04.020, PMID: 28450156

[ref58] Guo J. U. Agarwal V. Guo H. Bartel D. P. (2014). Expanded identification and characterization of mammalian circular RNAs. Genome Biol. 15, 1–14. doi: 10.1186/s13059-014-0409-z PMC4165365 25070500

[ref59] Gupta N. Jadhav S. Tan K.-L. Saw G. Mallilankaraman K. B. Dheen S. T. (2020). miR-142-3p regulates BDNF expression in activated rodent microglia through its target CAMK2A. Front. Cell. Neurosci. 14:132. doi: 10.3389/fncel.2020.00132, PMID: 32508597 PMC7253665

[ref60] Han B. Zhang Y. Zhang Y. Bai Y. Chen X. Huang R.. (2018). Novel insight into circular RNA HECTD1 in astrocyte activation via autophagy by targeting MIR142-TIPARP: implications for cerebral ischemic stroke. Autophagy 14, 1164–1184. doi: 10.1080/15548627.2018.1458173, PMID: 29938598 PMC6103660

[ref61] Hanan M. Simchovitz A. Yayon N. Vaknine S. Cohen-Fultheim R. Karmon M.. (2020). A Parkinson's disease Circ RNA s resource reveals a link between circ SLC 8A1 and oxidative stress. EMBO Mol. Med. 12:e11942. doi: 10.15252/emmm.202013551, PMID: 32715657 PMC7507321

[ref62] Hansen T. B. Jensen T. I. Clausen B. H. Bramsen J. B. Finsen B. Damgaard C. K.. (2013). Natural RNA circles function as efficient microRNA sponges. Nature 495, 384–388. doi: 10.1038/nature11993, PMID: 23446346

[ref63] Hansen M. Rubinsztein D. C. Walker D. W. (2018). Autophagy as a promoter of longevity: insights from model organisms. Nat. Rev. Mol. Cell Biol. 19, 579–593. doi: 10.1038/s41580-018-0033-y, PMID: 30006559 PMC6424591

[ref64] Harati R. Hammad S. Tlili A. Mahfood M. Mabondzo A. Hamoudi R. (2022). miR-27a-3p regulates expression of intercellular junctions at the brain endothelium and controls the endothelial barrier permeability. PLoS One 17:e0262152. doi: 10.1371/journal.pone.0262152, PMID: 35025943 PMC8758013

[ref65] Huang J.-L. Qin M.-C. Zhou Y. Xu Z.-H. Yang S.-M. Zhang F.. (2018). Comprehensive analysis of differentially expressed profiles of Alzheimer’s disease associated circular RNAs in an Alzheimer’s disease mouse model. Aging (Albany NY) 10, 253–265. doi: 10.18632/aging.101387, PMID: 29448241 PMC5842852

[ref66] Huang S. Yang B. Chen B. Bliim N. Ueberham U. Arendt T.. (2017b). The emerging role of circular RNAs in transcriptome regulation. Genomics 109, 401–407. doi: 10.1016/j.ygeno.2017.06.005 28655641

[ref67] Huang W. Zeng C. Hu S. Wang L. Liu J. (2019). ATG3, a target of miR-431-5p, promotes proliferation and invasion of colon cancer via promoting autophagy. Cancer Manag. Res. 11, 10275–10285. doi: 10.2147/CMAR.S226828, PMID: 31849517 PMC6911302

[ref68] Huang R. Zhang Y. Han B. Bai Y. Zhou R. Gan G.. (2017a). Circular RNA HIPK2 regulates astrocyte activation via cooperation of autophagy and ER stress by targeting MIR124–2HG. Autophagy 13, 1722–1741. doi: 10.1080/15548627.2017.1356975, PMID: 28786753 PMC5640207

[ref69] Huangfu L. Liang H. Wang G. Su X. Li L. Du Z.. (2016). miR-183 regulates autophagy and apoptosis in colorectal cancer through targeting of UVRAG. Oncotarget 7, 4735–4745. doi: 10.18632/oncotarget.6732, PMID: 26717041 PMC4826239

[ref70] Jiang Q. Su D.-Y. Wang Z.-Z. Liu C. Sun Y.-N. Cheng H.. (2021). Retina as a window to cerebral dysfunction following studies with circRNA signature during neurodegeneration. Theranostics 11, 1814–1827. doi: 10.7150/thno.51550, PMID: 33408783 PMC7778582

[ref71] Jiménez-Ortega R. F. Ramírez-Salazar E. G. Parra-Torres A. Y. Muñoz-Montero S. A. Rangel-Escareňo C. Salido-Guadarrama I.. (2017). Identification of microRNAs in human circulating monocytes of postmenopausal osteoporotic Mexican-mestizo women: a pilot study. Exp. Ther. Med. 14, 5464–5472. doi: 10.3892/etm.2017.5260, PMID: 29285077 PMC5740757

[ref72] Junn E. Lee K.-W. Jeong B. S. Chan T. W. Im J.-Y. Mouradian M. M. (2009). Repression of α-synuclein expression and toxicity by microRNA-7. Proc. Natl. Acad. Sci. 106, 13052–13057. doi: 10.1073/pnas.0906277106, PMID: 19628698 PMC2722353

[ref73] Kahl A. Blanco I. Jackman K. Baskar J. Milaganur Mohan H. Rodney-Sandy R.. (2018). Cerebral ischemia induces the aggregation of proteins linked to neurodegenerative diseases. Sci. Rep. 8, 1–8. doi: 10.1038/s41598-018-21063-z 29426953 PMC5807442

[ref74] Keita M. Mcintyre K. Rodden L. N. Schadt K. Lynch D. R. (2022). Friedreich ataxia: clinical features and new developments. Neurodegener. Dis. Manag. 12, 267–283. doi: 10.2217/nmt-2022-0011, PMID: 35766110 PMC9517959

[ref75] Klionsky D. J. Petroni G. Amaravadi R. K. Baehrecke E. H. Ballabio A. Boya P.. (2021). Autophagy in major human diseases. EMBO J. 40:e108863. doi: 10.15252/embj.2021108863, PMID: 34459017 PMC8488577

[ref76] Kondo M. A. Mohan A. Mather K. A. (2020). Going around in circles: deciphering the role of circular RNAs in neurodegenerative disease. Curr. Opin. Psychiatry 33, 141–147. doi: 10.1097/YCO.0000000000000582, PMID: 31895158

[ref77] Kong F. Lv Z. Wang L. Zhang K. Cai Y. Ding Q.. (2021). RNA-sequencing of peripheral blood circular RNAs in Parkinson disease. Medicine 100:e25888. doi: 10.1097/MD.0000000000025888, PMID: 34114985 PMC8202568

[ref78] Koutnikova H. Campuzano V. Foury F. Dollé P. Cazzalini O. Koenig M. (1997). Studies of human, mouse and yeast homologues indicate a mitochondrial function for frataxin. Nat. Genet. 16, 345–351. doi: 10.1038/ng0897-345, PMID: 9241270

[ref79] Kramer M. C. Liang D. Tatomer D. C. Gold B. March Z. M. Cherry S.. (2015). Combinatorial control of drosophila circular RNA expression by intronic repeats, hnRNPs, and SR proteins. Genes Dev. 29, 2168–2182. doi: 10.1101/gad.270421.115, PMID: 26450910 PMC4617980

[ref80] Kumar L. Jadiya P. Haque R. Shukla S. Nazir A. (2018). Functional characterization of novel circular RNA molecule, circzip-2 and its synthesizing gene zip-2 in C. elegans model of Parkinson’s disease. Mol. Neurobiol. 55, 6914–6926. doi: 10.1007/s12035-018-0903-5, PMID: 29363043

[ref81] Kwiatkowski J. R. T. Bosco D. Leclerc A. Tamrazian E. Vanderburg C. Russ C.. (2009). Mutations in the FUS/TLS gene on chromosome 16 cause familial amyotrophic lateral sclerosis. Science 323, 1205–1208. doi: 10.1126/science.1166066, PMID: 19251627

[ref82] Lee J.-A. (2009). Autophagy in neurodegeneration: two sides of the same coin. BMB Rep. 42, 324–330. doi: 10.5483/BMBRep.2009.42.6.324, PMID: 19558789

[ref83] Lee J.-A. Beigneux A. Ahmad S. T. Young S. G. Gao F.-B. (2007). ESCRT-III dysfunction causes autophagosome accumulation and neurodegeneration. Curr. Biol. 17, 1561–1567. doi: 10.1016/j.cub.2007.07.029, PMID: 17683935

[ref84] Li G. Chen T. Zhu Y. Xiao X. Bu J. Huang Z. (2018). MiR-103 alleviates autophagy and apoptosis by regulating SOX2 in LPS-injured PC12 cells and SCI rats. Iran. J. Basic Med. Sci. 21:292. doi: 10.22038/ijbms.2018.25980.6392, PMID: 29511496 PMC5817173

[ref85] Li Y. Fan H. Sun J. Ni M. Zhang L. Chen C.. (2020b). Circular RNA expression profile of Alzheimer’s disease and its clinical significance as biomarkers for the disease risk and progression. Int. J. Biochem. Cell Biol. 123:105747. doi: 10.1016/j.biocel.2020.105747, PMID: 32315771

[ref86] Li X. Feng Y. Wang X.-X. Truong D. Wu Y.-C. (2020a). The critical role of SIRT1 in Parkinson’s disease: mechanism and therapeutic considerations. Aging Dis. 11, 1608–1622. doi: 10.14336/AD.2020.0216, PMID: 33269110 PMC7673849

[ref87] Li S. Li L. Li J. Liang X. Song C. ZOU, Y (2022a). miR-203, fine-tunning neuroinflammation by juggling different components of NF-κB signaling. J. Neuroinflammation 19, 1–16. doi: 10.14336/AD.2020.0216 35413928 PMC9006621

[ref88] Li Y. Wang F. Teng P. Ku L. Chen L. Feng Y.. (2022c). Accurate identification of circRNA landscape and complexity reveals their pivotal roles in human oligodendroglia differentiation. Genome Biol. 23, 1–27. doi: 10.1186/s13059-022-02621-1 35130952 PMC8819885

[ref89] Li D. Yang Y. Li Z.-Q. Li L.-C. Zhu X.-H. (2019a). Circular RNAs: from biogenesis and function to diseases. Chin. Med. J. 132, 2457–2464. doi: 10.1097/CM9.0000000000000465, PMID: 31651510 PMC6831080

[ref90] Li T. Zhang H. Wang Z. Gao S. Zhang X. Zhu H.. (2022b). The regulation of autophagy by the miR-199a-5p/p62 axis was a potential mechanism of small cell lung cancer cisplatin resistance. Cancer Cell Int. 22, 1–17. doi: 10.1016/j.biocel.2020.105747 35292022 PMC8922820

[ref91] Li Y. Zhang G. Wu B. Yang W. Liu Z. (2019b). miR-199a-5p represses protective autophagy and overcomes chemoresistance by directly targeting DRAM1 in acute myeloid leukemia. J. Oncol. 2019, 1–16. doi: 10.1155/2019/5613417, PMID: 31636666 PMC6766143

[ref92] Liu X. Hua F. Yang D. Lin Y. Zhang L. Ying J.. (2022). Roles of neuroligins in central nervous system development: focus on glial neuroligins and neuron neuroligins. J. Transl. Med. 20, 1–19. doi: 10.1186/s12967-022-03625-y 36088343 PMC9463862

[ref93] Liu Z. Yang J. Fang Q. Shao H. Yang D. Sun J.. (2021). MiRNA-199a-5p targets WNT2 to regulate depression through the CREB/BDNF signaling in hippocampal neuron. Brain Behav. 11:e02107. doi: 10.1002/brb3.2107 34333859 PMC8413827

[ref94] Lo I. Hill J. Vilhjálmsson B. J. Kjems J. (2020). Linking the association between circRNAs and Alzheimer’s disease progression by multi-tissue circular RNA characterization. RNA Biol. 17, 1789–1797. doi: 10.1080/15476286.2020.1783487, PMID: 32618510 PMC7714474

[ref95] Lu Y. Gao J. Zhang S. Gu J. Lu H. Xia Y.. (2018). miR-142-3p regulates autophagy by targeting ATG16L1 in thymic-derived regulatory T cell (tTreg). Cell Death Dis. 9, 1–10. doi: 10.1038/s41419-018-0298-2 29459719 PMC5833855

[ref96] Lu Y. Tan L. Wang X. (2019). Circular HDAC9/microRNA-138/Sirtuin-1 pathway mediates synaptic and amyloid precursor protein processing deficits in Alzheimer’s disease. Neurosci. Bull. 35, 877–888. doi: 10.1007/s12264-019-00361-0, PMID: 30887246 PMC6754481

[ref97] Lukiw W. J. (2013). Circular RNA (circRNA) in Alzheimer's disease (AD). Front. Genet. 4:307. doi: 10.3389/fgene.2013.00307, PMID: 24427167 PMC3875874

[ref98] Luo D. Singh N. N. Singh R. N. (2022). Internal introns promote Backsplicing to generate circular RNAs from spinal muscular atrophy gene. Genes 13:1145. doi: 10.3390/genes13071145, PMID: 35885927 PMC9323214

[ref99] Lynch C. (2020). World Alzheimer report 2019: attitudes to dementia, a global survey: public health: engaging people in ADRD research. Alzheimers Dement. 16:e038255. doi: 10.1002/alz.038255

[ref100] Marfil-Marin E. Santamaría-Olmedo M. Perezgrovas-Saltijeral A. Valdes-Flores M. Ochoa-Morales A. Jara-Prado A.. (2021). circRNA regulates dopaminergic synapse, MAPK, and Long-term depression pathways in Huntington disease. Mol. Neurobiol. 58, 6222–6231. doi: 10.1007/s12035-021-02536-1, PMID: 34476673

[ref101] Martí E. Pantano L. Bañez-Coronel M. Llorens F. Miñones-Moyano E. Porta S.. (2010). A myriad of miRNA variants in control and Huntington’s disease brain regions detected by massively parallel sequencing. Nucleic Acids Res. 38, 7219–7235. doi: 10.1093/nar/gkq575, PMID: 20591823 PMC2978354

[ref102] Martinez B. Peplow P. V. (2021). Altered microRNA expression in animal models of Huntington’s disease and potential therapeutic strategies. Neural Regen. Res. 16:2159. doi: 10.4103/1673-5374.310673, PMID: 33818488 PMC8354140

[ref103] Mcmillan K. J. Murray T. K. Bengoa-Vergniory N. Cordero-Llana O. Cooper J. Buckley A.. (2017). Loss of microRNA-7 regulation leads to α-synuclein accumulation and dopaminergic neuronal loss in vivo. Mol. Ther. 25, 2404–2414. doi: 10.1016/j.ymthe.2017.08.017, PMID: 28927576 PMC5628933

[ref104] Memczak S. Jens M. Elefsinioti A. Torti F. Krueger J. Rybak A.. (2013). Circular RNAs are a large class of animal RNAs with regulatory potency. Nature 495, 333–338. doi: 10.1038/nature11928, PMID: 23446348

[ref105] Meng L. Liu S. Ding P. Chang S. Sang M. (2020). Circular RNA ciRS-7 inhibits autophagy of ESCC cells by functioning as miR-1299 sponge to target EGFR signaling. J. Cell. Biochem. 121, 1039–1049. doi: 10.1002/jcb.29339, PMID: 31490018

[ref106] Miao H. Miao C. Han J. Li N. (2020). Downregulation of miR-200a protects mouse leydig cells against triptolide by triggering autophagy. Drug Des. Devel. Ther. 14, 4845–4854. doi: 10.2147/DDDT.S269236, PMID: 33204070 PMC7667511

[ref107] Miller J. W. Urbinati C. R. Teng-Umnuay P. Stenberg M. G. Byrne B. J. Thornton C. A.. (2000). Recruitment of human muscleblind proteins to (CUG) n expansions associated with myotonic dystrophy. EMBO J. 19, 4439–4448. doi: 10.1093/emboj/19.17.4439, PMID: 10970838 PMC302046

[ref108] Min S.-W. Cho S.-H. Zhou Y. Schroeder S. Haroutunian V. Seeley W. W.. (2010). Acetylation of tau inhibits its degradation and contributes to tauopathy. Neuron 67, 953–966. doi: 10.1016/j.neuron.2010.08.044, PMID: 20869593 PMC3035103

[ref109] Mizushima N. (2007). Autophagy: process and function. Genes Dev. 21, 2861–2873. doi: 10.1101/gad.1599207, PMID: 18006683

[ref110] Nguyen D. K. Thombre R. Wang J. (2019). Autophagy as a common pathway in amyotrophic lateral sclerosis. Neurosci. Lett. 697, 34–48. doi: 10.1016/j.neulet.2018.04.006, PMID: 29626651 PMC6170747

[ref111] Nilsson P. Loganathan K. Sekiguchi M. Matsuba Y. Hui K. Tsubuki S.. (2013). Aβ secretion and plaque formation depend on autophagy. Cell Rep. 5, 61–69. doi: 10.1016/j.celrep.2013.08.042, PMID: 24095740

[ref112] Nilsson P. Saido T. C. (2014). Dual roles for autophagy: degradation and secretion of Alzheimer's disease Aβ peptide. BioEssays 36, 570–578. doi: 10.1002/bies.201400002, PMID: 24711225 PMC4316186

[ref113] Nixon R. A. (2007). Autophagy, amyloidogenesis and Alzheimer disease. J. Cell Sci. 120, 4081–4091. doi: 10.1242/jcs.019265, PMID: 18032783

[ref114] Nixon R. A. (2013). The role of autophagy in neurodegenerative disease. Nat. Med. 19, 983–997. doi: 10.1038/nm.3232, PMID: 23921753

[ref115] Nixon R. A. Wegiel J. Kumar A. Yu W. H. Peterhoff C. Cataldo A.. (2005). Extensive involvement of autophagy in Alzheimer disease: an immuno-electron microscopy study. J. Neuropathol. Exp. Neurol. 64, 113–122. doi: 10.1093/jnen/64.2.113, PMID: 15751225

[ref116] Oakes J. A. Davies M. C. Collins M. O. (2017). TBK1: a new player in ALS linking autophagy and neuroinflammation. Mol. Brain 10, 1–10. doi: 10.1186/s13041-017-0287-x 28148298 PMC5288885

[ref117] Oddo S. Caccamo A. Smith I. F. Green K. N. Laferla F. M. (2006). A dynamic relationship between intracellular and extracellular pools of Aβ. Am. J. Pathol. 168, 184–194. doi: 10.2353/ajpath.2006.050593, PMID: 16400022 PMC1592652

[ref118] Ottesen E. W. Singh R. N. (2020). Characteristics of circular RNAs generated by human survival motor neuron genes. Cell. Signal. 73:109696. doi: 10.1016/j.cellsig.2020.109696, PMID: 32553550 PMC7387165

[ref119] Paloczi J. Varga Z. V. Hasko G. Pacher P. (2018). Neuroprotection in oxidative stress-related neurodegenerative diseases: role of endocannabinoid system modulation. Antioxid. Redox Signal. 29, 75–108. doi: 10.1089/ars.2017.7144, PMID: 28497982 PMC5984569

[ref120] Park H. B. Lee S. M. Lee J. S. Park M. S. Park K. I. Namgung R.. (2010). Survival analysis of spinal muscular atrophy type I. Korean J. Pediatr. 53, 965–970. doi: 10.3345/kjp.2010.53.11.965, PMID: 21218019 PMC3012277

[ref121] Pascual M. Vicente M. Monferrer L. Artero R. (2006). The Muscleblind family of proteins: an emerging class of regulators of developmentally programmed alternative splicing. Differentiation 74, 65–80. doi: 10.1111/j.1432-0436.2006.00060.x, PMID: 16533306

[ref122] Peggion C. Scalcon V. Massimino M. L. Nies K. Lopreiato R. Rigobello M. P.. (2022). SOD1 in ALS: taking stock in pathogenic mechanisms and the role of glial and muscle cells. Antioxidants 11:614. doi: 10.3390/antiox11040614, PMID: 35453299 PMC9032988

[ref123] Piwecka M. Glažar P. Hernandez-Miranda L. R. Memczak S. Wolf S. A. Rybak-Wolf A.. (2017). Loss of a mammalian circular RNA locus causes miRNA deregulation and affects brain function. Science 357:eaam8526. doi: 10.1126/science.aam8526, PMID: 28798046

[ref124] Priller J. Scherzer C. R. Faber P. W. Macdonald M. E. Yong A. B. (1997). Frataxin gene of Friedreich's ataxia is targeted to mitochondria. Ann. Neurol. 42, 265–269. doi: 10.1002/ana.410420222, PMID: 9266741

[ref125] Quatrana A. Morini E. Tiano F. Vancheri C. Panarello L. Romano S.. (2022). Hsa-miR223-3p circulating level is upregulated in Friedreich’s ataxia and inversely associated with HCLS1 associated protein X-1, HAX-1. Hum. Mol. Genet. 31, 2010–2022. doi: 10.1093/hmg/ddac005, PMID: 35015850

[ref126] Querzfurth H. (2010). Review article. Mechanism of disease Alzheimer’disease. N. Engl. J. Med. 362, 329–344. doi: 10.1056/NEJMra0909142 20107219

[ref127] Ramesh N. Pandey U. B. (2017). Autophagy dysregulation in ALS: when protein aggregates get out of hand. Front. Mol. Neurosci. 10:263. doi: 10.3389/fnmol.2017.00263, PMID: 28878620 PMC5572252

[ref128] Ravanidis S. Bougea A. Karampatsi D. Papagiannakis N. Maniati M. Stefanis L.. (2021). Differentially expressed circular RNAs in peripheral blood mononuclear cells of patients with Parkinson's disease. Mov. Disord. 36, 1170–1179. doi: 10.1002/mds.28467, PMID: 33433033 PMC8248110

[ref129] Ray A. K. Dubois J. C. Gruber R. C. Guzik H. M. Gulinello M. E. Perumal G.. (2017). Loss of G as6 and A xl signaling results in extensive axonal damage, motor deficits, prolonged neuroinflammation, and less remyelination following cuprizone exposure. Glia 65, 2051–2069. doi: 10.1002/glia.23214, PMID: 28925029 PMC5643251

[ref130] Rea S. L. Ventura N. Johnson T. E. (2007). Relationship between mitochondrial electron transport chain dysfunction, development, and life extension in Caenorhabditis elegans. PLoS Biol. 5:e259. doi: 10.1371/journal.pbio.0050259, PMID: 17914900 PMC1994989

[ref131] Robinson M. Lee B. Y. Hane F. T. (2017). Recent progress in Alzheimer’s disease research, part 2: genetics and epidemiology. J. Alzheimers Dis. 57, 317–330. doi: 10.3233/JAD-161149, PMID: 28211812 PMC5366246

[ref132] Roser A.-E. Gomes L. C. Halder R. Jain G. Maass F. Tönges L.. (2018). miR-182-5p and miR-183-5p act as GDNF mimics in dopaminergic midbrain neurons. Mol. Ther. Nucleic Acids 11, 9–22. doi: 10.1016/j.omtn.2018.01.005, PMID: 29858093 PMC5849806

[ref133] Roshan R. Shridhar S. Sarangdhar M. A. Banik A. Chawla M. Garg M.. (2014). Brain-specific knockdown of miR-29 results in neuronal cell death and ataxia in mice. RNA 20, 1287–1297. doi: 10.1261/rna.044008.113, PMID: 24958907 PMC4105753

[ref134] Rudnick N. D. Griffey C. J. Guarnieri P. Gerbino V. Wang X. Piersaint J. A.. (2017). Distinct roles for motor neuron autophagy early and late in the SOD1G93A mouse model of ALS. Proc. Natl. Acad. Sci. 114, E8294–E8303. doi: 10.1073/pnas.1704294114, PMID: 28904095 PMC5625902

[ref135] Rusten T. E. Stenmark H. (2009). How do ESCRT proteins control autophagy? J. Cell Sci. 122, 2179–2183. doi: 10.1242/jcs.050021, PMID: 19535733

[ref136] Rybak-Wolf A. Stottmeister C. Glažar P. Jens M. Pino N. Giusti S.. (2015). Circular RNAs in the mammalian brain are highly abundant, conserved, and dynamically expressed. Mol. Cell 58, 870–885. doi: 10.1016/j.molcel.2015.03.027, PMID: 25921068

[ref137] Saido T. Leissring M. A. (2012). Proteolytic degradation of amyloid β-protein. Cold Spring Harb. Perspect. Med. 2:a006379. doi: 10.1101/cshperspect.a006379, PMID: 22675659 PMC3367539

[ref138] Salas I. H. Burgado J. Allen N. J. (2020). Glia: victims or villains of the aging brain? Neurobiol. Dis. 143:105008. doi: 10.1016/j.nbd.2020.105008, PMID: 32622920

[ref139] Salmena L. Poliseno L. Tay Y. Kats L. Pandolfi P. P. (2011). A ceRNA hypothesis: the Rosetta stone of a hidden RNA language? Cells 146, 353–358. doi: 10.1016/j.cell.2011.07.014, PMID: 21802130 PMC3235919

[ref140] Sang Q. Liu X. Wang L. Qi L. Sun W. Wang W.. (2018). CircSNCA downregulation by pramipexole treatment mediates cell apoptosis and autophagy in Parkinson’s disease by targeting miR-7. Aging (Albany NY) 10, 1281–1293. doi: 10.18632/aging.101466, PMID: 29953413 PMC6046232

[ref141] Sansa A. Hidalgo I. Miralles M. P. De La Fuente S. Perez-Garcia M. J. Munell F.. (2021). Spinal muscular atrophy autophagy profile is tissue-dependent: differential regulation between muscle and motoneurons. Acta Neuropathol. Commun. 9, 1–15. doi: 10.1186/s40478-021-01223-5 34217376 PMC8254901

[ref142] Santos R. Lefevre S. Sliwa D. Seguin A. Camadro J.-M. Lesuisse E. (2010). Friedreich ataxia: molecular mechanisms, redox considerations, and therapeutic opportunities. Antioxid. Redox Signal. 13, 651–690. doi: 10.1089/ars.2009.3015, PMID: 20156111 PMC2924788

[ref143] Schmucker S. Argentini M. Carelle-Calmels N. Martelli A. Puccio H. (2008). The in vivo mitochondrial two-step maturation of human frataxin. Hum. Mol. Genet. 17, 3521–3531. doi: 10.1093/hmg/ddn244, PMID: 18725397

[ref144] Schrank B. Götz R. Gunnersen J. M. Ure J. M. Toyka K. V. Smith A. G.. (1997). Inactivation of the survival motor neuron gene, a candidate gene for human spinal muscular atrophy, leads to massive cell death in early mouse embryos. Proc. Natl. Acad. Sci. 94, 9920–9925. doi: 10.1073/pnas.94.18.9920, PMID: 9275227 PMC23295

[ref145] Seco-Cervera M. González-Rodríguez D. Ibáñez-Cabellos J. Peiró-Chova L. González-Cabo P. García-López E.. (2017). Circulating miR-323-3p is a biomarker for cardiomyopathy and an indicator of phenotypic variability in Friedreich’s ataxia patients. Sci. Rep. 7, 1–12. doi: 10.1038/s41598-017-04996-9 28701783 PMC5507909

[ref146] Sekar S. Liang W. S. (2019). Circular RNA expression and function in the brain. Non-coding RNA Res. 4, 23–29. doi: 10.1016/j.ncrna.2019.01.001, PMID: 30891534 PMC6404376

[ref147] Shang Y. Liu Q. Wang L. Qiu X. Chen Y. An J. (2021). microRNA-146a-5p negatively modulates PM2. 5 caused inflammation in THP-1 cells via autophagy process. Environ. Pollut. 268:115961. doi: 10.1016/j.envpol.2020.115961, PMID: 33160737

[ref148] Shi Z. Chen T. Yao Q. Zheng L. Zhang Z. Wang J.. (2017). The circular RNA ci RS-7 promotes APP and BACE 1 degradation in an NF-κB-dependent manner. FEBS J. 284, 1096–1109. doi: 10.1111/febs.14045, PMID: 28296235

[ref149] Shioya M. Obayashi S. Tabunoki H. Arima K. Saito Y. Ishida T.. (2010). Aberrant microRNA expression in the brains of neurodegenerative diseases: miR-29a decreased in Alzheimer disease brains targets neurone navigator 3. Neuropathol. Appl. Neurobiol. 36, 320–330. doi: 10.1111/j.1365-2990.2010.01076.x, PMID: 20202123

[ref150] Si X. Cao D. Chen J. Nie Y. Jiang Z. Chen M. Y.. (2018). miR-23a downregulation modulates the inflammatory response by targeting ATG12-mediated autophagy. Mol. Med. Rep. 18, 1524–1530. doi: 10.3892/mmr.2018.9081, PMID: 29845275 PMC6072189

[ref151] Simon D. Seznec H. Gansmuller A. Carelle N. Weber P. Metzger D.. (2004). Friedreich ataxia mouse models with progressive cerebellar and sensory ataxia reveal autophagic neurodegeneration in dorsal root ganglia. J. Neurosci. 24, 1987–1995. doi: 10.1523/JNEUROSCI.4549-03.2004, PMID: 14985441 PMC6730414

[ref152] Singh N. N. O'leary C. A. Eich T. Moss W. Singh R. N. (2022). Structural context of a critical exon of spinal muscular atrophy gene. Front. Mol. Biosci. 702. doi: 10.3389/fmolb.2022.928581 PMC9283826 35847983

[ref153] Singh R. N. Seo J. Singh N. N. (2020). RNA in spinal muscular atrophy: therapeutic implications of targeting. Expert Opin. Ther. Targets 24, 731–743. doi: 10.1080/14728222.2020.1783241, PMID: 32538213 PMC7529864

[ref154] Smrt R. D. Szulwach K. E. Pfeiffer R. L. Li X. Guo W. Pathania M.. (2010). MicroRNA miR-137 regulates neuronal maturation by targeting ubiquitin ligase mind bomb-1. Stem Cells 28, 1060–1070. doi: 10.1002/stem.431, PMID: 20506192 PMC3140955

[ref155] Steffan J. S. (2010). Does huntingtin play a role in selective macroautophagy? Cell Cycle 9, 3401–3413. doi: 10.4161/cc.9.17.12718, PMID: 20703094 PMC3047613

[ref156] Sturrock A. Leavitt B. R. (2010). The clinical and genetic features of Huntington disease. J. Geriatr. Psychiatry Neurol. 23, 243–259. doi: 10.1177/0891988710383573, PMID: 20923757

[ref157] Sugiyama T. Taniguchi K. Matsuhashi N. Tajirika T. Futamura M. Takai T.. (2016). MiR-133b inhibits growth of human gastric cancer cells by silencing pyruvate kinase muscle-splicer polypyrimidine tract-binding protein 1. Cancer Sci. 107, 1767–1775. doi: 10.1111/cas.13091, PMID: 27696637 PMC5198967

[ref158] Sutedja N. A. Veldink J. H. Fischer K. Kromhout H. Heederik D. Huisman M. H.. (2009). Exposure to chemicals and metals and risk of amyotrophic lateral sclerosis: a systematic review. Amyotroph. Lateral Scler. 10, 302–309. doi: 10.3109/17482960802455416, PMID: 19922117

[ref159] Tang C. Ou J. Kou L. Deng J. Luo S. (2020). Circ_016719 plays a critical role in neuron cell apoptosis induced by I/R via targeting miR-29c/Map2k6. Mol. Cell. Probes 49:101478. doi: 10.1016/j.mcp.2019.101478, PMID: 31698040

[ref160] Tian R. Wu B. Fu C. Guo K. (2020). miR-137 prevents inflammatory response, oxidative stress, neuronal injury and cognitive impairment via blockade of Src-mediated MAPK signaling pathway in ischemic stroke. Aging (Albany NY) 12, 10873–10895. doi: 10.18632/aging.103301, PMID: 32496209 PMC7346022

[ref161] Torgovnick A. Schiavi A. Testi R. Ventura N. (2010). A role for p53 in mitochondrial stress response control of longevity in C. elegans. Exp. Gerontol. 45, 550–557. doi: 10.1016/j.exger.2010.02.007, PMID: 20172019

[ref162] Vance C. Rogelj B. Hortobágyi T. De Vos K. J. Nishimura A. L. Sreedharan J.. (2009). Mutations in FUS, an RNA processing protein, cause familial amyotrophic lateral sclerosis type 6. Science 323, 1208–1211. doi: 10.1126/science.1165942, PMID: 19251628 PMC4516382

[ref163] Verduci L. Tarcitano E. Strano S. Yarden Y. Blandino G. (2021). CircRNAs: role in human diseases and potential use as biomarkers. Cell Death Dis. 12, 1–12. doi: 10.1038/s41419-021-03743-3 33976116 PMC8113373

[ref164] Verheijen B. M. Pasterkamp R. J. (2017). Commentary: FUS affects circular RNA expression in murine embryonic stem cell-derived motor neurons. Front. Mol. Neurosci. 10:412. doi: 10.3389/fnmol.2017.00412, PMID: 29311805 PMC5732946

[ref165] Vicencio E. Beltrán S. Labrador L. Manque P. Nassif M. Woehlbier U. (2020). Implications of selective autophagy dysfunction for ALS pathology. Cells 9:381. doi: 10.3390/cells9020381, PMID: 32046060 PMC7072226

[ref166] Wang W. Lv R. Zhang J. Liu Y. (2021b). circSAMD4A participates in the apoptosis and autophagy of dopaminergic neurons via the miR-29c-3p-mediated AMPK/mTOR pathway in Parkinson's disease. Mol. Med. Rep. 24, 1–10. doi: 10.3892/mmr.2021.12179 PMC8170871 34080649

[ref167] Wang Y. Mo Y. Peng M. Zhang S. Gong Z. Yan Q.. (2022). The influence of circular RNAs on autophagy and disease progression. Autophagy 18, 240–253. doi: 10.1080/15548627.2021.1917131, PMID: 33904341 PMC8942425

[ref168] Wang H. Sun G. Xu P. Lv J. Zhang X. Zhang L.. (2021a). Circular RNA TMEM87A promotes cell proliferation and metastasis of gastric cancer by elevating ULK1 via sponging miR-142-5p. J. Gastroenterol. 56, 125–138. doi: 10.1007/s00535-020-01744-1, PMID: 33155080

[ref169] Wang X. Tan L. Lu Y. Peng J. Zhu Y. Zhang Y.. (2015). MicroRNA-138 promotes tau phosphorylation by targeting retinoic acid receptor alpha. FEBS Lett. 589, 726–729. doi: 10.1016/j.febslet.2015.02.001, PMID: 25680531

[ref170] Wang Z. Xu P. Chen B. Zhang Z. Zhang C. Zhan Q.. (2018). Identifying circRNA-associated-ceRNA networks in the hippocampus of Aβ1-42-induced Alzheimer's disease-like rats using microarray analysis. Aging (Albany NY) 10, 775–788. doi: 10.18632/aging.101427, PMID: 29706607 PMC5940119

[ref171] Wei Y. Hong D. Zang A. Wang Z. Yang H. Zhang P.. (2021). miR-183 enhances autophagy of GC cells by targeted inhibition of mTOR. Ann. Clin. Lab. Sci. 51, 837–843., PMID: 34921037

[ref172] Wilusz J. E. Sharp P. A. (2013). A circuitous route to noncoding RNA. Science 340, 440–441. doi: 10.1126/science.1238522, PMID: 23620042 PMC4063205

[ref173] Wu Q. Wang H. Liu L. Zhu K. Yu W. Guo J. (2020). Hsa_circ_0001546 acts as a miRNA-421 sponge to inhibit the chemoresistance of gastric cancer cells via ATM/Chk2/p53-dependent pathway. Biochem. Biophys. Res. Commun. 521, 303–309. doi: 10.1016/j.bbrc.2019.10.117, PMID: 31668372

[ref174] Xie K. Colgan L. A. Dao M. T. Muntean B. S. Sutton L. P. Orlandi C.. (2016). NF1 is a direct G protein effector essential for opioid signaling to ras in the striatum. Curr. Biol. 26, 2992–3003. doi: 10.1016/j.cub.2016.09.010, PMID: 27773571 PMC5121064

[ref175] Xu L. Ji H. Jiang Y. Cai L. Lai X. Wu F.. (2020). Exosomes derived from CircAkap7-modified adipose-derived mesenchymal stem cells protect against cerebral ischemic injury. Front. Cell Dev. Biol. 1066 doi: 10.3389/fcell.2020.569977 PMC7573549 33123535

[ref176] Xu Z. Li Z. Wang W. Xia Y. He Z. Li B.. (2019). MIR-1265 regulates cellular proliferation and apoptosis by targeting calcium binding protein 39 in gastric cancer and, thereby, impairing oncogenic autophagy. Cancer Lett. 449, 226–236. doi: 10.1016/j.canlet.2019.02.026, PMID: 30779944

[ref177] Yamazaki Y. Takahashi T. Hiji M. Kurashige T. Izumi Y. Yamawaki T.. (2010). Immunopositivity for ESCRT-III subunit CHMP2B in granulovacuolar degeneration of neurons in the Alzheimer's disease hippocampus. Neurosci. Lett. 477, 86–90. doi: 10.1016/j.neulet.2010.04.038, PMID: 20420883

[ref178] Yang Y. Klionsky D. J. (2020). Autophagy and disease: unanswered questions. Cell Death Differ. 27, 858–871. doi: 10.1038/s41418-019-0480-9, PMID: 31900427 PMC7206137

[ref179] Yang H. Wang H. Shang H. Chen X. Yang S. Qu Y.. (2019). Circular RNA circ_0000950 promotes neuron apoptosis, suppresses neurite outgrowth and elevates inflammatory cytokines levels via directly sponging miR-103 in Alzheimer’s disease. Cell Cycle 18, 2197–2214. doi: 10.1080/15384101.2019.1629773, PMID: 31373242 PMC6738533

[ref180] Yang J.-H. Zhang R.-J. Lin J.-J. Cao M.-C. Wang Q. Cui H.-X.. (2018). The differentially expressed circular RNAs in the substantia nigra and corpus striatum of Nrf2-knockout mice. Cell. Physiol. Biochem. 50, 936–951. doi: 10.1159/000494478, PMID: 30355941

[ref185] Yang Q. Zhao Q. Yin Y. (2019). miR‑133b is a potential diagnostic biomarker for Alzheimer’s disease and has a neuroprotective role. Exp. Ther. Med. 18, 2711–2718. 31572518 10.3892/etm.2019.7855 PMC6755445

[ref181] Ye Z. Fang B. Pan J. Zhang N. Huang J. Xie C.. (2017). miR-138 suppresses the proliferation, metastasis and autophagy of non-small cell lung cancer by targeting Sirt1. Oncol. Rep. 37, 3244–3252. doi: 10.3892/or.2017.5619, PMID: 28498463 PMC5442395

[ref182] You X. Vlatkovic I. Babic A. Will T. Epstein I. Tushev G.. (2015). Neural circular RNAs are derived from synaptic genes and regulated by development and plasticity. Nat. Neurosci. 18, 603–610. doi: 10.1038/nn.3975, PMID: 25714049 PMC4376664

[ref183] Youdim M. B. (2010). Why do we need multifunctional neuroprotective and neurorestorative drugs for Parkinson's and Alzheimer's diseases as disease modifying agents. Exp. Neurobiol. 19, 1–14. doi: 10.5607/en.2010.19.1.1, PMID: 22110336 PMC3214798

[ref184] Yu W. H. Cuervo A. M. Kumar A. Peterhoff C. M. Schmidt S. D. Lee J.-H.. (2005). Macroautophagy—a novel β-amyloid peptide-generating pathway activated in Alzheimer's disease. J. Cell Biol. 171, 87–98. doi: 10.1083/jcb.200505082, PMID: 16203860 PMC2171227

[ref186] Zarei S. Carr K. Reiley L. Diaz K. Guerra O. Altamirano P. F.. (2015). A comprehensive review of amyotrophic lateral sclerosis. Surg. Neurol. Int. 6:171. doi: 10.4103/2152-7806.169561, PMID: 26629397 PMC4653353

[ref187] Zeng Z. Fei L. Yang J. Zuo J. Huang Z. Li H. (2021). MiR-27a-3p targets GLP1R to regulate differentiation, autophagy, and release of inflammatory factors in pre-osteoblasts via the AMPK signaling pathway. Front. Genet. 12, 783352–783352. doi: 10.3389/fgene.2021.783352, PMID: 35069685 PMC8766720

[ref188] Zhang M. An C. Gao Y. Leak R. K. Chen J. Zhang F. (2013). Emerging roles of Nrf2 and phase II antioxidant enzymes in neuroprotection. Prog. Neurobiol. 100, 30–47. doi: 10.1016/j.pneurobio.2012.09.003, PMID: 23025925 PMC3623606

[ref189] Zhang M. Bian Z. (2021). The emerging role of circular RNAs in Alzheimer’s disease and Parkinson’s disease. Front. Aging Neurosci. 13:691512. doi: 10.3389/fnagi.2021.691512, PMID: 34322012 PMC8311738

[ref190] Zhang S. Chen S. Liu A. Wan J. Tang L. Zheng N.. (2018). Inhibition of BDNF production by MPP+ through up-regulation of miR-210-3p contributes to dopaminergic neuron damage in MPTP model. Neurosci. Lett. 675, 133–139. doi: 10.1016/j.neulet.2017.10.014, PMID: 29030221

[ref191] Zhang G.-Y. Wang J. Jia Y.-J. Han R. Li P. Zhu D.-N. (2015). MicroRNA-9 promotes the neuronal differentiation of rat bone marrow mesenchymal stem cells by activating autophagy. Neural Regen. Res. 10:314. doi: 10.4103/1673-5374.143439, PMID: 25883633 PMC4392682

[ref192] Zhang J. Wang P. Wan L. Xu S. Pang D. (2017). The emergence of noncoding RNAs as Heracles in autophagy. Autophagy 13, 1004–1024. doi: 10.1080/15548627.2017.1312041, PMID: 28441084 PMC5486373

[ref193] Zhang X. Wang S. Wang H. Cao J. Huang X. Chen Z.. (2019). Circular RNA circNRIP1 acts as a microRNA-149-5p sponge to promote gastric cancer progression via the AKT1/mTOR pathway. Mol. Cancer 18, 20–24. doi: 10.1186/s12943-018-0935-5, PMID: 30717751 PMC6360801

[ref194] Zhang Y. Zhao Y. Liu Y. Wang M. Yu W. Zhang L. (2020). Exploring the regulatory roles of circular RNAs in Alzheimer’s disease. Transl. Neurodegener. 9, 1–8. doi: 10.1186/s40035-019-0179-3, PMID: 32951610 PMC7504624

[ref195] Zhao Y. Alexandrov P. N. Jaber V. Lukiw W. J. (2016). Deficiency in the ubiquitin conjugating enzyme UBE2A in Alzheimer’s disease (AD) is linked to deficits in a natural circular miRNA-7 sponge (circRNA; ciRS-7). Genes 7:116. doi: 10.3390/genes7120116, PMID: 27929395 PMC5192492

[ref196] Zhao Y. Jaber V. R. Lukiw W. J. (2022). Current advances in our understanding of circular RNA (circRNA) in Alzheimer’s disease (AD); the potential utilization of synthetic circRNAs as a therapeutic strategy in the clinical management of AD. Front. Drug Discov. 19

[ref197] Zheng S. Clabough E. B. Sarkar S. Futter M. Rubinsztein D. C. Zeitlin S. O. (2010). Deletion of the huntingtin polyglutamine stretch enhances neuronal autophagy and longevity in mice. PLoS Genet. 6:e1000838. doi: 10.1371/journal.pgen.1000838, PMID: 20140187 PMC2816686

[ref198] Zhou X. Jiang L. Fan G. Yang H. Wu L. Huang Y.. (2019). Role of the ciRS-7/miR-7 axis in the regulation of proliferation, apoptosis and inflammation of chondrocytes induced by IL-1β. Int. Immunopharmacol. 71, 233–240. doi: 10.1016/j.intimp.2019.03.037, PMID: 30925324

[ref199] Zhou Z. Zhang Y. Gao J. Hao X. Shan C. Li J.. (2021b). Circular RNAs act as regulators of autophagy in cancer. Mol. Ther. Oncolytics 21, 242–254. doi: 10.1016/j.omto.2021.04.007, PMID: 34095462 PMC8142048

[ref200] Zhou Q. Zhang M.-M. Liu M. Tan Z.-G. Qin Q.-L. Jiang Y.-G. (2021a). LncRNA XIST sponges miR-199a-3p to modulate the Sp1/LRRK2 signal pathway to accelerate Parkinson’s disease progression. Aging (Albany NY) 13, 4115–4137. doi: 10.18632/aging.202378, PMID: 33494069 PMC7906184

[ref201] Zingale V. D. Gugliandolo A. Mazzon E. (2021). MiR-155: an important regulator of Neuroinflammation. Int. J. Mol. Sci. 23:90. doi: 10.3390/ijms23010090, PMID: 35008513 PMC8745074

